# Neuroprotective Effect of *Nor*-Prenylated Acylphloroglucinols from *Hypericum perforatum* L. (St John’s Wort) in the MPTP-Induced Zebrafish Model

**DOI:** 10.3390/ijms26073096

**Published:** 2025-03-27

**Authors:** Wuyang Liu, Peng Zhao, Yihan Liu, Xiangyan Meng, Jinyan Xie, Junmian Tian, Jinming Gao

**Affiliations:** Shaanxi Key Laboratory of Natural Products & Chemical Biology, College of Chemistry & Pharmacy, Northwest A&F University, Yangling 712100, China; liuwuyang94@nwafu.edu.cn (W.L.); zpxp@nwafu.edu.cn (P.Z.); yhliu@nwafu.edu.cn (Y.L.); mengxy@nwafu.edu.cn (X.M.); jinyanxie@nwafu.edu.cn (J.X.)

**Keywords:** *Hypericum perforatum* L., *nor*-prenylated acylphloroglucinols, polysubstituted cyclohexanone, neuroprotective effect, Parkinson’s disease

## Abstract

*Hypericum perforatum* L. (St John’s wort) has been widely studied and used for antidepressant treatment, as well as, rarely, featuring in studies on its chemical composition for Parkinson’s disease (PD) treatment. Five new *nor*-prenylated acylphloroglucinols with a cyclohexanone core, norperforatums A–E (**1**–**5**), together with four known analogs [(2*R*,3*R*,4*S*,6*R*)-3-methyl-4,6-di(3-methyl-2-butenyl)-2-(2-methyl-1-oxopropyl)-3-(4-methyl-3-pentenyl)cyclohexanone (**6**), hyperscabrin B (**7**), (2*R*,3*R*,4*S*,6*R*)-6-methoxycarbonyl-3-methyl-4,6-di(3-methyl-2-butenyl)-2-(2-methyl-1-oxopropyl)-3-(4-methyl-3-pentenyl)cyclohexanone (**8**), and hyperscabin K (**9**)], were isolated from the aerial parts of *H. perforatum*. The structures and absolute configurations of the new compounds were characterized by multiple spectroscopic means, including nuclear magnetic resonance (NMR), high-resolution electrospray ionization mass spectrometry (HR-ESI-MS), ultraviolet visible absorption spectroscopy (UV), infrared spectroscopy (IR), calculated electronic circular dichroism (ECD) data, and X-ray signal crystal diffraction. In addition, the efficacy of these isolations was evaluated against 1-methyl-4-phenyl-1,2,3,6-tetrahydropyridine (MPTP)-induced PD in zebrafish larvae. Compound **9** had the best therapeutic effect, by significantly increasing the total distance traveled and the mean speed of movement in PD dyskinesia zebrafish larvae. Moreover, it enhanced superoxide dismutase (SOD) activity and inhibited reactive oxygen species (ROS) production in a dose-dependent manner. These results suggest that compound **9** may have ameliorative effects on PD symptoms by inhibiting oxidative stress. This study provides new insights into the treatment of *H. perforatum* for PD.

## 1. Introduction

*Hypericum perforatum* L., also known as St John’s wort, is a traditional Chinese medicine applied to treat mild and moderate depression in many countries [1]. The first polycyclic polyprenylated acylphloroglucinol (PPAP), hyperforin, was isolated and identified in 1971 from *H. perforatum,* and is now considered to be the main constituent of its antidepressant activity [2,3]. The genus *Hypericum* has been extensively studied, and more than 1200 PPAPs with diverse skeletons are reported with the in-depth exploration of chemical constituents, of which the majority are the typical bicylo[3,3,1]nonane-2,4,9-trione core PPAPs [4,5]. *Nor*-polyprenylated acylphloroglucinols are derivatives of the cleavage of bicyclic PAPs (BPAPs), where the backbone is replaced by a cyclohexanone core. These *nor*-PPAP derivatives are capable of undergoing further reactions, including cyclization and oxidation, to form more intricate carbon skeletons [6,7,8]. These kinds of metabolites exhibit a wide range of biological activities, such as anti-inflammation [7], hepatoprotective activity [9], lipid-lowering activity [6], and neuroprotective effects [10,11]. PPAPs have attracted particular interest for their neuroprotective activities in recent years [4,12,13,14].

Parkinson’s disease (PD) is the second most common neurodegenerative disease, characterized by a progressive loss of dopaminergic neurons in the substantia nigra pars compacta (SNpc), ultimately leading to motor dysfunction [15]. The zebrafish (*Danio rerio*) has demonstrated significant strengths as a model organism in the study of human diseases, particularly in the neurodegeneration, neurobehavioral, neurodevelopmental, toxicology, and drug discovery areas [16]. Its advantages include low cost, ease of maintenance, rapid evolution, a high number of transparent embryos, and ease of experimental manipulation by a variety of methods, including genetic methods [17]. In addition, as a vertebrate, the research results obtained for zebrafish may have greater mammalian applicability compared to genetic models of invertebrate models such as *Drosophila melanogaster* and *Caenorhabditis elegans*. In zebrafish, 1-methyl-4-phenyl-1,2,3,6-tetrahydropyridine (MPTP) is one of the most commonly used neurotoxins for inducing PD models, with a mechanism of toxic action that is highly similar to that of humans [18]. MPTP is able to induce degradation of dopamine (DA) neurons, and to reduce dopamine, norepinephrine, and serotonin levels in the brain. Motor dysfunction was observed in the MPTP-induced zebrafish PD model, as evidenced by the slowed swimming speed and abnormal swimming behaviors, which were consistent with the symptoms of motor retardation in PD patients [16]. MPTP is converted to its active form, MPP+, by the metabolism of monoamine oxidase B (MAO-B) in glial cells [19]. Since the chemical structure of MPP+ is similar to that of dopamine, it is then recognized by the dopamine transporter protein (DAT) and transported into dopamine (DA) neurons. Inside the neurons, MPP+ accumulates predominantly in the mitochondria, where it blocks the normal function of the mitochondrial electron transport chain by binding to the mitochondrial complex I [20]. This binding not only disrupts the subsequent functioning of the mitochondrial electron transport chain, but it also leads to a lack of energy production, as evidenced by a decrease in adenosine triphosphate (ATP) synthesis, and an increase in the production of reactive oxygen species (ROS) [16,21].

**Figure 1:**
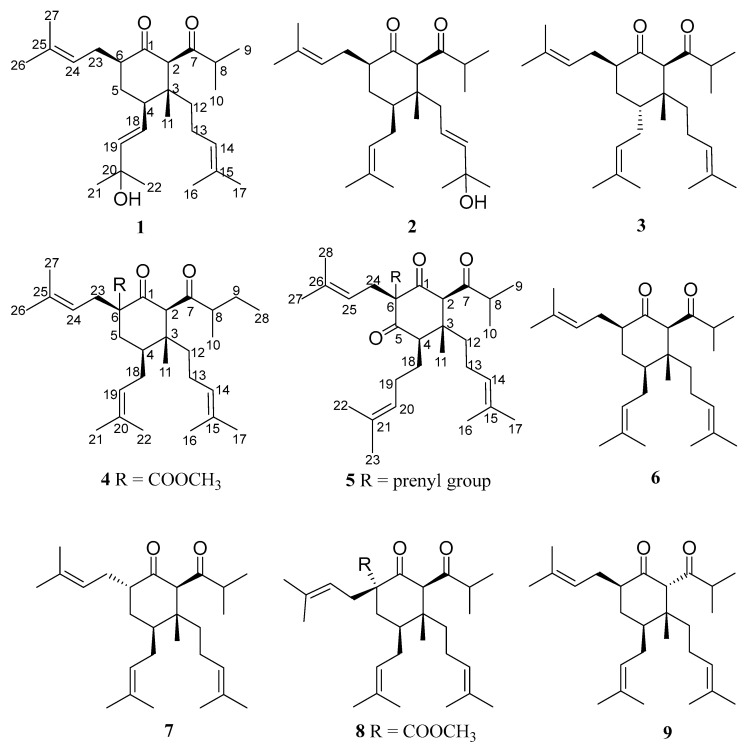
Structures of compounds **1**–**9**.

Our group has previously isolated and characterized a large number of bioactive components from the genus *Hypericum* [5,11,14,22,23,24,25,26]. The present chemical study led to the isolation and characterization of nine *nor*-PPAPs from *H. perforatum*, five of which were undescribed (Figure 1). The structures of norperforatums A–E (**1**–**5**) were determined by spectroscopic analyses, including high-resolution electrospray ionization mass spectrometry (HR-ESI-MS), infrared spectroscopy (IR), ultraviolet visible absorption spectroscopy (UV), nuclear magnetic resonance (NMR), and electronic circular dichroism (ECD) calculation, as well as an X-ray signal crystal diffraction. In addition, we screened these isolations for their role in ameliorating 1-methyl-4-phenyl-1,2,3,6-tetrahydropyridine (MPTP)-induced PD in zebrafish larvae.

## 2. Results

### 2.1. Structural Elucidation

**Figure 2:**
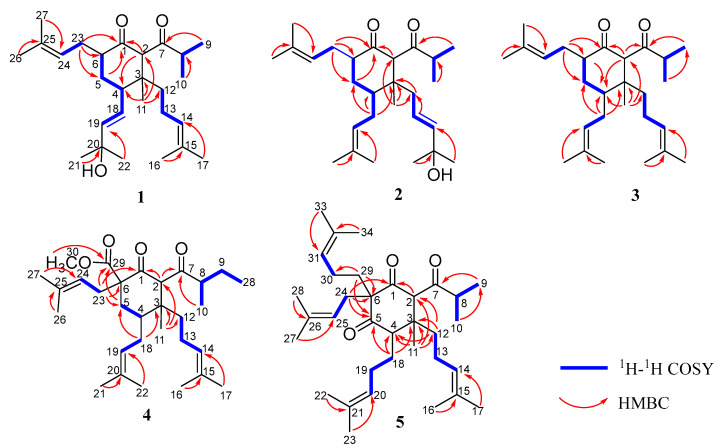
^1^H–^1^H COSY and key HMBC correlations of compounds **1**–**5**.

**Figure 3:**
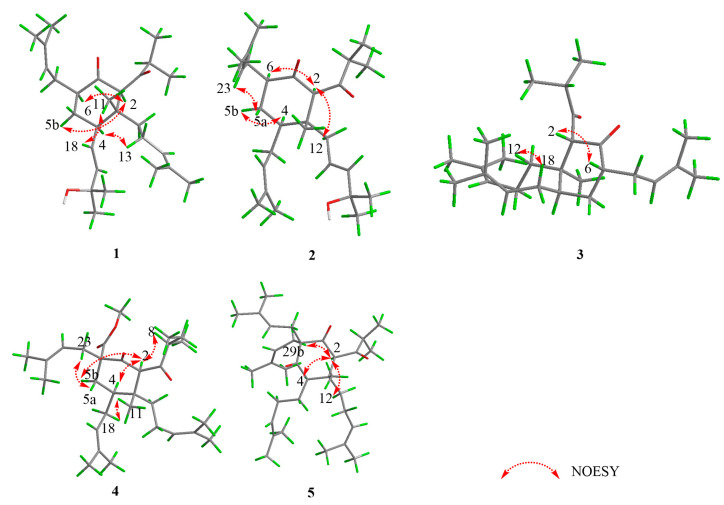
NOESY correlations of compounds **1**–**5**.

**Figure 4:**
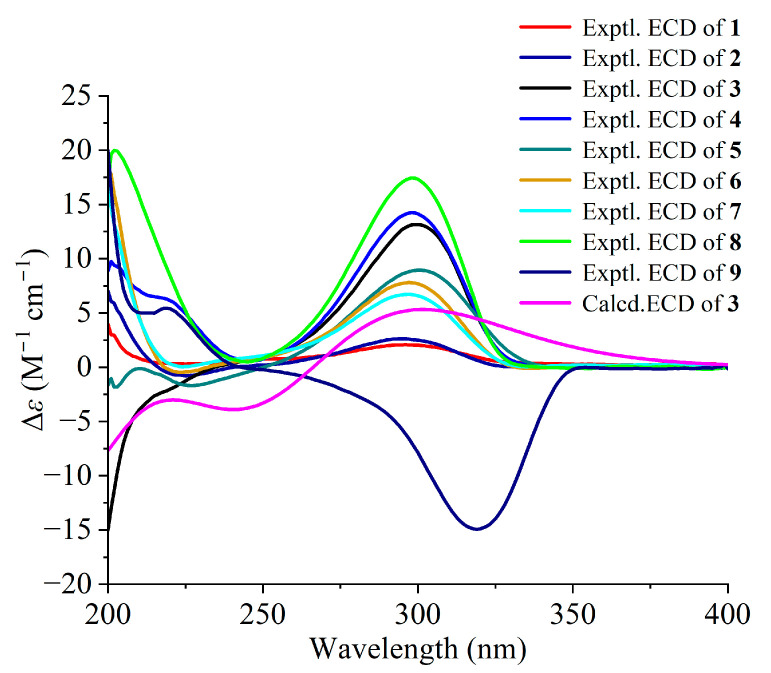
Calculated and experimental CD spectra of compounds **1**–**9**.

**Figure 5:**
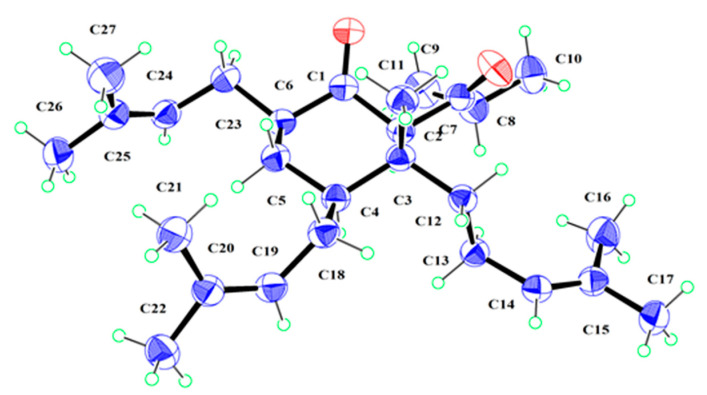
ORTEP drawing of compound **6**. Blue represents carbon atom, green represents hydrogen atom, red represents oxygen atom.

Norperforatum A (**1**) was isolated as a colorless oil with the molecular formula C_27_H_44_O_3_ deduced from the HR-ESI-MS data at *m*/*z* 439.3166 [M + Na]^+^ (calcd. for C_27_H_44_O_3_Na^+^, 439.3183). The degrees of unsaturation were calculated and shown to be six. The ^1^H NMR spectrum showed four olefinic protons (*δ*_H_ 5.74, 1H, d, *J* = 15.6 Hz; 5.53, 1H, dd, *J* = 15.6, 8.8 Hz; 5.07, 1H, t, *J* = 6.5 Hz; 4.93, 1H, t, *J* = 6.5 Hz), an isopropyl group (*δ*_H_ 2.50, 1H, m; 1.06, 3H, d, *J* = 6.8 Hz; 1.05, 3H, d, *J* = 6.8 Hz), and seven singlet methyls (*δ*_H_ 1.68, 1.63, 1.60, 1.56, 1.32, 1.32, 1.02, s). The ^13^C NMR spectrum showed two carbonyl groups (*δ*_C_ 210.8, 209.7) (Table 1 and Table 2). The above description occupied five degrees of unsaturation, of which norperforatum A belongs to the monocyclic polyprenylated acylphloroglucinol (MPAP) derivative [7]. A comparison of the 1D and 2D NMR data of norperforatum A to **6** revealed that their structures were very similar (Figures S1–S11). The HMBC correlations from H_3_-21 to C-20 (*δ*_C_ 70.9) and C-19 (*δ*_C_ 140.6), together with ^1^H-^1^H COSY correlations of H-4/H-18/H-19, confirmed that the 3-methylbut-1-en-3-ol group was connected at the C-4 position in norperforatum A, instead of an isoprenyl group (Figure 2). The NOESY interactions of H-2/H-6 indicated that H-2 and H-6 were in the same orientation, arranged for *α*-orientation. The NOESY interactions of H_3_-11/H_2_-18 and H-4/H_2_-13 indicated that H_3_-11 and the isoprenyl group at the C-4 position, located in the same orientation, were arranged for *β*-orientation (Figure 3). In addition, the experimental CD data of norperforatum A were similar to those of **6,** with the positive Cotton effect around 205 nm and 300 nm (Figure 4). The absolute configuration of norperforatum A was identified as **6**, and the absolute configuration was characterized by X-ray crystal diffraction (Cu K*α*) (Figure 5). Thus, the absolute configuration of norperforatum A was confirmed to be 2*R*,3*R*,4*R*,6*R*.

Norperforatum B (**2**) shared the same molecular formula as C_27_H_44_O_3_, based on their HR-ESI-MS data at *m*/*z* 439.3174 [M + Na]^+^ (calcd. for C_27_H_44_O_3_Na^+^, 439.3183), of which the degrees of unsaturation were calculated to be six. The planar structure of norperforatum B was similar to norperforatum A by detailed comparison and analysis of the 1D and 2D NMR spectra (Figures S12–S22), in which the main difference was the 3-methylbut-1-en-3-ol group, which was connected at C-12 position in norperforatum B, rather than at the C-4 position. These inferences were confirmed by the HMBC and ^1^H–^1^H COSY correlations (Figure 2). The NOESY correlations of H-6/H-2/H_2_-12, H-4/H_b_-5 and H_a_-5/H-23 revealed that norperforatum B had the same relative configuration to that of **6** (Figure 3). In addition, the experimental CD curve also resembled those of **6**, indicating that the absolute configuration of norperforatum B was consistent with **6** (Figure 4). The absolute configuration of norperforatum B was determined to be 2*R*,3*R*,4*S*,6*R*.

Norperforatum C (**3**), a colorless oil, had a molecular formula of C_27_H_44_O_2_, according to its HR-ESI-MS data at *m*/*z* 401.3409 [M + H]^+^ (calcd. for C_27_H_45_O_2_^+^, 401.3414), indicating six degrees of unsaturation. The ^1^H NMR spectrum revealed the characteristic signals of three olefinic protons (*δ*_H_ 5.00, 1H, overlapped; 4.96, 1H, overlapped; 4.92, 1H, overlapped), an isopropyl group (*δ*_H_ 2.36, 1H, m; 0.96, 3H, overlapped; 0.96, 3H, overlapped), and seven singlet methyls (*δ*_H_ 1.67, 1.65, 1.61, 1.61, 1.56, 1.54, 0.96, s) (Table 1). The characteristic signals of two carbonyl carbons (*δ*_C_ 212.4, 211.3) and six olefinic carbons (*δ*_C_ 133.9, 132.8, 131.7, 123.7, 123.2, 121.0) were observed from the ^13^C NMR spectrum (Table 2). The careful analyses of the 2D NMR data of norperforatum C revealed that the planar structure was identical to that of **6** (Figure 2 and Figures S23–S33). However, the deviation of chemical shifts in the ^13^C NMR spectrum, and a negative absorption around 210 nm in the CD spectrum, suggested that the absolute configuration of norperforatum C was changed. The NOESY correlation of H-2 with H-6 suggested that two protons were in the same orientation, assigned as the *α*-orientation. Moreover, the NOESY correlation of H_2_-12 with H_2_-18 indicated that isoprenyl groups at C-3 and C-4 were co-oriented (Figure 3). The ECD calculation indicated that norperforatum C was established to be the C-4 epimer of **6**. Moreover, the calculated spectrum was consistent with that of the experimental spectrum (Figure 4). Finally, the absolute configuration of norperforatum C was established as 2*R*,3*R*,4*R*,6*R*.

Norperforatum D (**4**) was obtained a colorless oil. The molecular formula of C_30_H_48_O_4_ was determined by its HR-ESI-MS data at *m*/*z* 473.3623 [M + H]^+^ (calcd. for C_30_H_49_O_4_^+^, 473.3625), indicating seven degrees of unsaturation. The ^1^H NMR data exhibited three olefinic protons (*δ*_H_ 5.13, 1H, overlapped; 5.05, 1H, overlapped; 4.93, 1H, t, *J* = 7.1 Hz), seven singlet methyls (*δ*_H_ 1.74, 1.68, 1.63, 1.59, 1.55, 1.55, 0.96, s), and a methoxy group (*δ*_H_ 3.76, s) (Table 1). The ^13^C NMR and HSQC spectra indicated thirty carbon signals, including eight quaternary carbon (two carbonyl carbons, an ester carbonyl carbon, and three olefinic carbons), six methylenes, six methines (three olefinic carbons), a methoxy group, and nine methyls. Norperforatum D shared the same carbon skeleton of compound **8,** as shown through the analysis of the 1D and 2D NMR spectra (Figures S34–S44) [27], with the major differences of an *s*-butyl group at C-7 (*δ*_H_ 0.88, t, *J* = 7.4 Hz; 1.06, d, *J* = 6.7 Hz; 1.25, m; 1.62, m; 2.37, m) instead of an *i*-Pr group (Table 1). This deduction was supported by the HMBC correlations from H_3_-28 to C-8/C-9 and from H_3_-10 to C-7, as well as the ^1^H–^1^H COSY correlations of H_3_-10/H-8/H_2_-9/H_3_-28 (Figure 2). The relative configuration of norperforatum D closely resembled that of **8**, as determined by the detailed NOESY analysis of H-4/H-2/H_b_-5, H_a_-5/H-23, and H_3_-11/H_2_-18 (Figure 3). Moreover, the stereo-center of the C-8 position cannot be determined, since it was in the side chain and easily rotated. Finally, the absolute configuration of norperforatum D was confirmed to be 2*R*,3*R*,4*S*,6*S* through a comparison of the CD spectrum to **8** (Figure 4).

Norperforatum E (**5**) possessed a molecular formula of C_34_H_54_O_3_ based on the HR-ESI-MS data (*m*/*z* 511.4126 [M + H]^+^, calcd. for C_34_H_55_O_3_^+^, 511.4146), indicating eight degrees of unsaturation. An analysis of the ^1^H and ^13^C NMR spectra showed the characteristic signals, including four olefinic protons (*δ*_H_ 4.91, t, *J* = 6.7 Hz; 4.95, t, *J* = 7.0 Hz; 5.02, t, *J* = 7.3 Hz; 5.12, t, *J* = 7.8 Hz; *δ*_C_ 118.4, 122.6, 122.6, 123.6), three carbonyl carbons (*δ*_C_ 207.9, 210.2, 211.2), and an isopropyl group (*δ*_H_ 1.02, d, *J* = 6.6 Hz; 1.08, d, *J* = 6.6 Hz; 2.57, m; *δ*_C_ 17.7, 18.8, 42.5) (Table 1 and Table 2). The above description implied that norperforatum E was a 1,5-diketone monocyclic PPAP. The planar structure of norperforatum E was similar to **6**, except for the substituents at C-4 and C-6, as evidenced by the HMBC correlations and ^1^H–^1^H COSY cross-peaks (Figure 2 and Figures S45–S55). The same relative configuration of norperforatum E as compound **6** was disclosed by the NOESY correlations of H-4/H-2/H_2_-12 and H-2/H_b_-29 (Figure 3). Moreover, the CD spectrum of norperforatum E resembled that of **6** (Figure 4). Thus, the absolute configuration of norperforatum E was confirmed to be 2*R*,3*R*,4*R*,6*R*.

Four known compounds, (2*R*,3*R*,4*S*,6*R*)-3-methyl-4,6-di(3-methyl-2-butenyl)-2-(2-methyl-1-oxopropyl)-3-(4-methyl-3-pentenyl)cyclohexanone (**6**) [27], hyperscabrin B (**7**) [28], (2*R*,3*R*,4*S*,6*R*)-6-methoxycarbonyl-3-methyl-4,6-di(3-methyl-2-butenyl)-2-(2-methyl-1-oxopropyl)-3-(4-methyl-3-pentenyl)cyclohexanone (**8**) [27], and hyperscabin K (**9**) [29], were also isolated. These structures were identified based on their NMR spectra, as well as by comparing the NMR data to the reported literature.

### 2.2. Acute Toxicity Assessment of Compounds

**Figure 6:**
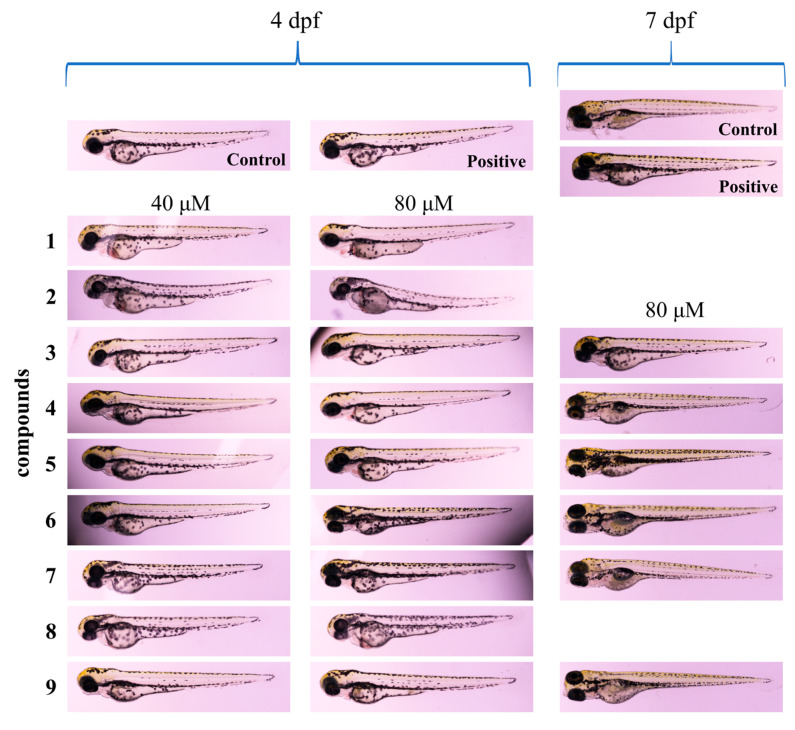
Zebrafish larvae morphology at 4 dpf (larvae exposed to 40 μM and 80 μM concentrations) and 7 dpf (larvae exposed to 80 μM concentration) during compounds toxicity testing. Scale bar: 1000 μm.

Given their high throughput and visualization advantages, zebrafish have been widely used in drug toxicology studies [30,31]. In order to determine that the administered concentrations for subsequent activity experiments were within a safe range, we assessed the acute toxicity of compounds **1**–**9** using zebrafish embryos. The results showed that zebrafish larvae exhibited different states after five consecutive days of exposure to different concentrations of compounds. Zebrafish larvae exposed to compounds **1**, **2**, and **8** (40 μM and 80 μM) for two consecutive days (4 dpf) exhibited significant pericardial edema, cardiac hemorrhage, and body bending (Figure 6). All larvae from these three groups died after 5 days (7 dpf) of continuous exposure. Compounds **3**–**7** and **9** in the 80 μM concentration range did not show significant toxicity to zebrafish larvae, which remained 100% alive and morphologically developed normally after 5 days of continuous dosing (Figure 6 and Figure S56). In addition, the positive control (1 mM levodopa methyl ester hydrochloride) we used was also safe.

### 2.3. Effect of Compounds on MPTP-Induced PD-like Dyskinesia

**Figure 7:**
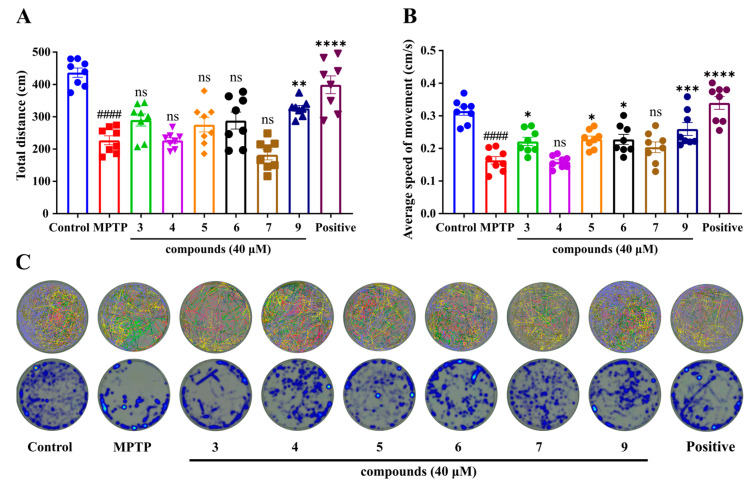
Effects of the isolated compounds on MPTP-induced locomotor impairments in zebrafish larvae. Total distance traveled (**A**), average speed (**B**), and track visualization image and heatmap image (**C**). The lines of different colors represent the movement trajectory of each larvae. The experimental groups included the control group, model group, treatment groups, and positive drug group (n = 8). #### *p* < 0.0001 vs. control (DMSO) group, * *p* < 0.05, ** *p* < 0.01, *** *p* < 0.001, **** *p* < 0.0001 vs. MPTP group; ns indicates no significance.

MPTP is a neurotoxin commonly used in zebrafish to induce Parkinson’s disease (PD), and it easily crosses the blood–brain barrier (BBB). After exposure to MPTP, zebrafish exhibit movement disorders, such as a slowed swimming speed and abnormal swimming behavior, which are similar to the motor retardation exhibited by humans [16]. We induced a PD zebrafish model using MPTP and assessed the effect of the compounds in ameliorating PD dyskinesia by analyzing their total swimming distance and average speed of movement. The results showed (Figure 7A,B) that the total swimming distance and the average speed of movement were significantly reduced and slowed down in MPTP-induced PD zebrafish compared to the control group. A total of 40 μM of compound **9** and the positive control drug markedly improved the total swimming distance and the average speed of movement of zebrafish larvae. The other compounds (compounds **3**, **4**, **5**, **6,** and **7**) did not show significant improvement. Figure 7C showed the movement trajectories and stay heatspots of zebrafish in each group.

### 2.4. Effect of Compound **9** on MPTP-Induced PD-like Dyskinesia

**Figure 8:**
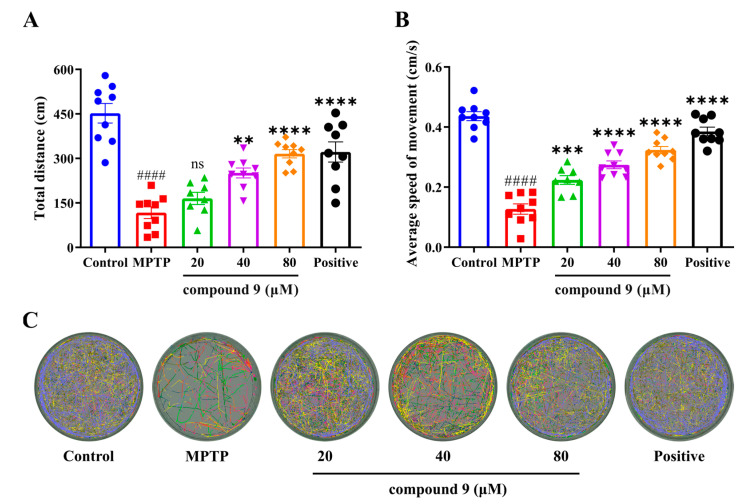
Effects of compound **9** at different concentrations on MPTP-induced locomotor impairments in zebrafish larvae. Total distance traveled (**A**), average speed (**B**), and track visualization image (**C**). The lines of different colors represent the movement trajectory of each larvae. The experimental groups included the control group, model group, treatment groups, and positive drug group (n = 9). #### *p* < 0.0001 vs. control (DMSO) group, ** *p* < 0.01, *** *p* < 0.001, **** *p* < 0.0001 vs. MPTP group; ns indicates no significance.

To further determine the effect of compound **9** on ameliorating PD symptoms, we tested the effects of different concentrations of compound **9** on the behavior of PD zebrafish larvae. The results showed (Figure 8A,B) that compound **9** at 40 and 80 μM significantly improved the total swimming distance and average swimming speed of PD zebrafish larvae, whereas compound **9** at 20 μM did not show significant effects. Figure 8C showed the movement trajectories of zebrafish in each group after treatment with different concentrations of compound **9**. These results indicated that compound **9** had an ameliorating effect on PD.

### 2.5. Effect of Compound **9** on Reactive Oxygen Species (ROS) Generation in PD Zebrafish Model

**Figure 9:**
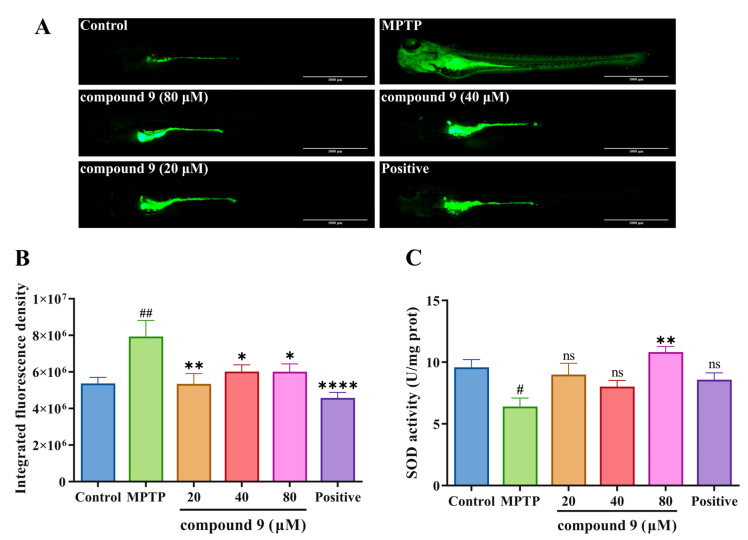
Effect of compound **9** on ROS levels and SOD activity in PD zebrafish model. (**A**) Fluorescence images of ROS generation in zebrafish larvae treated with DMSO, MPTP, different concentrations of compound **9**, positive drug. Scale bar, 1000 µm. (**B**) Quantification of the integrated fluorescence density of ROS levels in individual zebrafish larvae in A (n = 15–19). (**C**) SOD activity in larvae of different treatment groups (n = 3). # *p* < 0.05, ## *p* < 0.01 vs. control (DMSO) group, * *p* < 0.05, ** *p* < 0.01, **** *p* < 0.0001 vs. MPTP; ns indicates no significant difference.

MPTP can induce the accumulation of ROS production, and we further investigated the ability of compound **9** to inhibit ROS generation in vivo. Since the body of zebrafish larvae is transparent, the fluorescence after staining can be directly observed under a fluorescence microscope, which is convenient for characterization and quantification. Therefore, the fluorescent probe DCFH-DA can be directly utilized to stain zebrafish larvae to detect reactive oxygen species in vivo. Detecting the fluorescence of DCF will determine the level of intracellular ROS [32,33]. As shown in Figure 9A,B, compared to the control group, the larvae in the MPTP group showed obvious green fluorescence, indicating that a large amount of ROS was produced within the larvae under MPTP treatment, whereas the fluorescence signals in the larvae were weakened by the treatment with compound **9** and the positive drug, suggesting that the compound **9** and the positive drug were able to effectively inhibit the production of ROS. In addition, we examined the superoxide dismutase (SOD) activity in zebrafish larvae. The results showed that the SOD activity of the larvae in the MPTP group was significantly reduced compared to the control group. As the concentration of compound **9** increased, the SOD activity gradually recovered (Figure 9C). The above results indicated that compound **9** could increase the SOD activity of larvae and inhibit the MPTP-induced ROS production.

## 3. Discussion

PPAPs are a complex and diverse class of natural products mainly distributed in the genus *Hypericum*. The family, around 1200 PPAPs, has been categorized into monocyclic polyprenylated acylphloroglucinols (MPAPs), bicyclic polyprenylated acylphloroglucinols (BPAPs)—including types A and B depending on the relative position of the acyl group, *seco*-PPAPs, caged PPAPs, spirocyclic PPAPs, and intricate PPAPs through intramolecular [4 + 2] cycloadditions [4]. However, BPAPs with a major bicyclo[3.3.1]nonane-2,4,9-trione core could further undergo oxidative breakdown to obtain *nor*-polyprenylated acylphloroglucinols [7,34]. So far, only ten *nor*-polyprenylated acylphloroglucinols were found from *H. perforatum,* and the quality of the new compounds was very poor [10,34,35]. This work systematically studied the *nor*-polyprenylated acylphloroglucinols, which five new compounds (**1**–**5**) and four known ones (**6**–**9**) were isolated and determined by multiple separation methods and comprehensive analysis of NMR spectra, ECD calculations, and X-ray signal crystal diffraction. Compounds **1**–**9** are representatives of *nor*-polyprenylated acylphloroglucinols with a cyclohexanone core. Biogenetically, *nor*-polyprenylated acylphloroglucinols were proposed to be generated through the oxidative breakdown of hyperforin which could be derived from 2,4,6-trihydroxybenzophenone via a cascade of isopentenylation and intramolecular cyclization [34]. Compounds **1**–**9** were further formed by undergoing hydrogenation of the ester, decarboxylation, and oxidation of isoprenyl group [34,35]. Interestingly, the difference in the configuration at C-2 for hyperscabin K (**9**) reversed the optical rotation (OR) direction of the whole compound, of which the experimental CD curve around 320 nm and the OR direction ([*α*]^20^_D_ −128.1 (*c* 0.1 MeOH)) were complete opposites compared to the other compounds (Figure 4).

Toxicity studies are a very important element prior to activity evaluation. Zebrafish can be successfully employed to determine the toxicity of samples in early screening assays, often in a high throughput manner due to the short time required for analyses, transparency of the embryos, the short life cycle, high fertility, and genetic data similarity [36,37]. Extract of *H. perforatum* has attracted attention for its toxicity in the treatment of depression. Long-term treatment with *H. perforatum* during pregnancy or lactation causes severe histological damage to the liver and kidney in rats [38]. Compounds **1**–**9** are *nor*-polyprenylated acylphloroglucinols, with a cyclohexanone as the carbon skeleton, which was not tested for toxicity in zebrafish larvae in previous studies. Compounds **1**, **2,** and **8** showed significant toxicity in the concentration range of 10–80 μM (Figure 6), causing a marked pericardial edema, cardiac hemorrhage, and body bending. This study provides a new insight into the toxicity exploration of PPAPs. In the concentration range of 10–80 μM, compounds **1** and **2** exhibited significant toxicity compared to the other compounds. This property was probably due to the oxidation of the isoprenyl side chain connected at the C-4 and C-12 positions, which, in turn, substantially enhances the toxicity. The presence of the hydroxyl group might improve the solubility and bioavailability of the compounds, thereby increasing their toxicity [39]. In addition, the key difference between compounds **4** and **8** is the substituent at the C-7 position, where it is substituted with isopropyl at the C-7 position in compound **8**. The result was probably related to the substitution of the isopropyl group. Briefly, oxidation of the isoprenyl group significantly increases the toxicity of the compounds.

*H. perforatum* has been used in traditional medicine for centuries to treat several disorders, including minor burns, anxiety, and mild to moderate depression [40,41,42,43]. Its extracts are marketed as a dietary supplement in Europe, such as GNC St. John’s Wort Extract Capsules and Swisse Mood Tablets [44,45]. Moreover, it was reported that the extracts of *H. perforatum* had anti-PD and antiepileptic effects, but its compositions, mechanism, and clinical efficacy are yet to be elucidated [46,47,48]. Based on the above findings, compounds **1**–**9** were isolated and evaluated for anti-PD activity by MPTP-induced PD symptoms, and the results showed that compound **9** could improve PD-like dyskinesia compared to other compounds (Figure 7). Moreover, compound **9** significantly improved the PD-like locomotor impairments in zebrafish larvae across different batches (Figure 8 and Figure S57). These results were consistent with those described in the literature, in which symptoms of motor imbalance were alleviated after administration in the zebrafish PD model [49]. The configuration of C-2 changed in compound **9**, resulting in a reversal of its physicochemical properties, such as OR and CD, as well as affecting its bioactivity, compared to other compounds. This change might increase the affinity of the compound for the target proteins. It was reported that conformational changes in compounds might increase cell survival in the model of oxygen and glucose deprivation/deoxygenation or cell cytotoxicity [50,51,52]. Moreover, the configurations of the chiral carbons located at the C-2 and C-6 positions are able to change during the process of oxidative cleavage, but conformational change at C-6 position did not affect the activity of the compounds. Oxidative stress plays a critical role in the pathogenesis of PD and other neurodegenerative diseases [53]. ROS accumulation leads to oxidative damage to DNA and RNA, as well as impairing the neuronal function and structural integrity [54]. It has been reported that ROS levels were upregulated in the animal model of PD [55], which is in agreement with what we observed in the MPTP group. Importantly, compound **9** significantly inhibited MPTP-induced ROS production. Moreover, compound **9** could also improve levels of SOD. The results suggested that compound **9** might exert an anti-PD effect by inhibiting oxidative stress (Figure 9). The results indicated that *nor*-polyprenylated acylphloroglucinols of *H.perforatum* may be the material basis of anti-PD, and its in-depth mechanism needs to be further explored.

## 4. Materials and Methods

### 4.1. General Experimental Procedures

Optical rotations were measured with an Autopol III automatic polarimeter (Rudolph Research Analytical, Hackettstown, NJ, USA). UV and experimental CD spectra were obtained using a Chirascan spectrometer (Chirascan, Edinburgh, UK). A Bruker Tensor 27 and a Vertex70 FTIR spectrophotometer with KBr pellets were applied to obtain the IR spectra (Bruker, Ettlingen, Germany). NMR spectra were acquired on a Bruker Avance 400 MHz with tetramethylsilane (TMS) as the internal standard (Bruker, Kista, Sweden). A LC-30A + TripleTOF5600+ was used to obtain the HR-ESI-MS data (AB SCIEX, Singapore, Singapore). Semipreparative HPLC separations were performed with a shimadzu LC-20AP liquid chromatography system (Shimadzu, Kyoto, Japan). Silica gel (200–300 and 300–400 mesh, Qingdao Marine Chemical Co., Ltd., Qingdao, China), ODS RP-C18 (50 μm, YMC Co., Ltd., Kyoto, Japan), Sephadex LH-20 (40–70 μm, Amersham Pharmacia Biotech AB, Stockholm, Sweden) and reversed-phase (RP) C-18 semipreparative column (10 mm × 250 mm, 5 μm, YMC Co., Ltd., Kyoto, Japan) were employed to complete the separations and purifications of the samples. Organic solvents were obtained from Chron Chemicals Co., Ltd., Chengdu, China. Reactive oxygen detection kit was purchased from Beyotime Biotechnology (Shanghai, China). 

### 4.2. Plant Material

The aerial parts of *H. perforatum* were collected from Niubeiliang Mountain, Zhashui County, Shangluo City, Shaanxi Province, China (GPS: 108°59′35″ E, 33°50′15″ N), in August 2018. The plants were identified by Pro. Zhenhai Wu, School of Life Science, Northwest A&F University. The voucher specimen (no. 20180805HPL) was reserved in the Shaanxi Key Laboratory of Natural Products & Chemical Biology, Northwest A&F University.

### 4.3. Extraction and Isolation

The aerial portions of *H. perforatum* (100.0 kg) were powdered and extracted in three batches, with 95% EtOH (90 L × 3, each batch) by refluxing (50 °C, 3 h). Crude extract (11.8 kg) was obtained after the removal of the solutions in vacuum. The crude extract was suspended in water (50 L), and then partitioned with *n*-hexane (50 L × 3) and ethyl acetate (50 L × 3). The *n*-hexane extracts (1.0 kg) were chromatographed over a silica gel column with a gradient of petroleum ether-EtOAc (from 1:0 to 0:1, *v*/*v*) to obtain six fractions (A–F). Fr. B (283.0 g) was fractionated by a silica gel column using a gradient of petroleum ether-EtOAc (from 1:0 to 0:1, *v*/*v*) to obtain eight fractions (B1–B8). Fr. B5 (98.4 g) was isolated by a SephadexLH-20 column (CHCl_3_/MeOH, 1:1, *v*/*v*) to yield four fractions (B5a–B5d). Fr. B5c (16.0 g) was separated on a silica gel column eluted with *n*-hexane/dichloromethane (from 50:1 to 1:1, *v*/*v*), and was then subjected to an RP-C18 column (MeOH/H_2_O, 85:15 to 100:0, *v*/*v*) to yield five fractions (B5c1–B5c5). Fr. B5c3 (1.8 g) was applied to a silica gel column and ODS column to obtain compounds **3** (235.2 mg), **6** (200.3 mg), and **7** (15.9 mg). Fr. B5c3 (371.1 mg) and Fr. B5c5 (76.3 mg) were further isolated by semipreparative HPLC (MeOH:H_2_O, 95:5, *v*/*v*, 2 mL/min; MeOH:H_2_O, 98:2, *v*/*v*, 2 mL/min) to produce compounds **5** (50.2 mg) and **9** (20.7 mg). Fr. E (33.0 g) was chromatographed on an RP-C18 column with MeOH/H_2_O (70:30 to 100:0, *v*/*v*), resulting in three fractions (E1–E3). Fr. E3 (7.3 g) was repeatedly separated using the Sephadex LH-20 column (CHCl_3_/MeOH, 1:1, *v*/*v*) and silica gel column with *n*-hexane/dichloromethane (from 5:1 to 1:1, *v*/*v*) to obtain four fractions (E3a–E3d). Fr. E3a (317.0 mg) was purified by a silica gel column using petroleum ether/EtOAC (from 50:1 to 10:1, *v*/*v*) and semipreparative HPLC (MeOH:H_2_O, 90:10, *v*/*v*, 2 mL/min) to yield compounds **1** (5.0 mg) and **2** (4.7 mg). Fr. E3c (446.6 mg) was isolated by a silica gel CC (*n*-hexane/dichloromethane, from 5:1 to 1:1, *v*/*v*) and semipreparative HPLC (CH_3_CN:H_2_O, 90:10, *v*/*v*, 2 mL/min) to produce compounds **4** (6.0 mg) and **8** (107.2 mg).

Norperforatum A (**1**): colorless oil; [*α*]^20^_D_ +12.6 (*c* 0.035 MeOH); UV (MeOH) *λ*_max_ (log *ε*): 200 (4.50) nm; ECD (MeOH) *λ*_max_ (Δ*ε*): 296 (+1.50) nm; IR (KBr) *ν*_max_ 2967, 2925, 2868, 1714, 1453, 1372, 1232, 1120, 1047, 974 cm^−1^; 1D NMR data, see Table 1 and Table 2; HR-ESI-MS *m*/*z* 439.3166 [M + Na]^+^ (calcd. for C_27_H_44_O_3_Na^+^, 439.3183).

Norperforatum B (**2**): colorless oil; [*α*]^20^_D_ +20.1 (*c* 0.03 MeOH); UV (MeOH) *λ*_max_ (log *ε*): 200 (4.66) nm; ECD (MeOH) *λ*_max_ (Δ*ε*): 295 (+2.64) nm; IR (KBr) *ν*_max_ 2968, 2924, 2870, 1714, 1452, 1374, 1234, 973 cm^−1^; 1D NMR data, see Table 1 and Table 2; HR-ESI-MS *m*/*z* 439.3174 [M + Na]^+^ (calcd. for C_27_H_44_O_3_Na^+^, 439.3183).

Norperforatum C (**3**): colorless oil; [*α*]^20^_D_ +58.3 (*c* 0.07 MeOH); UV (MeOH) *λ*_max_ (log *ε*): 200 (4.74) nm; ECD (MeOH) *λ*_max_ (Δ*ε*): 299 (+13.68) nm; IR (KBr) *ν*_max_ 2969, 2925, 2874, 1713, 1451, 1378 cm^−1^; 1D NMR data, see Table 1 and Table 2; HR-ESI-MS *m*/*z* 401.3409 [M + H]^+^ (calcd. for C_27_H_45_O_2_^+^, 401.3414).

Norperforatum D (**4**): colorless oil; [*α*]^20^_D_ +77.6 (*c* 0.1 MeOH); UV (MeOH) *λ*_max_ (log *ε*): 200 (4.13) nm; ECD (MeOH) *λ*_max_ (Δ*ε*): 201 (+2.48) and 298 (+3.63) nm; IR (KBr) *ν*_max_ 3425, 2925, 1720, 1453, 1374, 1234 cm^−1^; 1D NMR data, see Table 1 and Table 2; HR-ESI-MS *m*/*z* 473.3623 [M + H]^+^ (calcd. for C_30_H_49_O_4_^+^, 473.3625).

Norperforatum E (**5**): colorless oil; [*α*]^20^_D_ +137.9 (*c* 0.1 MeOH); UV (MeOH) *λ*_max_ (log *ε*): 200 (4.45) nm; ECD (MeOH) *λ*_max_ (Δ*ε*): 300 (+8.94) nm; IR (KBr) *ν*_max_ 2968, 2923, 2873, 1713, 1449, 1376 cm^−1^; 1D NMR data, see Table 1 and Table 2; HR-ESI-MS *m*/*z* 511.4126 [M + H]^+^ (calcd. for C_34_H_55_O_3_^+^, 511.4146).

### 4.4. X-Ray Crystallographic Analysis

The crystals of compound **6**, monoclinic, were obtained at 4 °C in a solution of dichloromethane/methanol (1:1). A suitable crystal was selected and measured using a ROD, Synergy Custom system, and HyPix diffractometer. The crystallographic data have been submitted to the Cambridge Crystallographic Data Center (CCDC 2328908).

The crystal data are as follows (**6**): C_27_H_44_O_2_, *M_r_* = 400.65, monoclinic, obtained from dichloromethane, space group *P* 2_1_; *a* = 8.9605(3) Å, *b* = 10.2851(3) Å, *c* = 13.9455(4) Å, alpha = 90°, beta = 90.904(2)°, gamma = 90°, *V* = 1285.05(7) Å^3^, *Z* = 2, *μ* (Cu K*α*) = 0.477 mm^−1^, *D*_x_ = 1.035 1.083, and *F*(000) = 445.2; *T* = 150 K; crystal dimensions: 0.2 × 0.15 × 0.1 mm^3^; 13 132 reflections collected (9.88° ≤ 2Θ ≤ 149.36°), 5023 independent reflections (R_int_ = 0.0348, R_sigma_ = 0.0456). The final *R* indexes were 0.0864 (*I* > 2*σ*(*I*)). The final *wR*_2_ indexes were 0.2167 (*I* > 2*σ*(*I*)). The final *R* indexes were 0.0886 (all data). The final *wR*_2_ indexes were 0.2212 (all data). The good of fit on *F*^2^ was 1.083. Flack parameter = −0.1(3).

### 4.5. ECD Calculations

The conformational search of compound **3** was completed based on the MMFF94S force fields. Saved conformers were optimized using the density functional theory (DFT) method and the CPCM solvent model at the B3LYP/6–31(d,p) level. The time-dependent density functional theory (TDDFT) ECD calculations were performed at the Cam-B3LYP/Def2SVP level of theory in MeOH with the IEFPCM solvent model [56], in which the detailed CD calculations followed the previously published papers [57].

### 4.6. Acute Toxicity Assessment

The acute toxicity of all compounds was assessed in zebrafish embryos. Synchronized 2 days post-fertilization (dpf), zebrafish eggs were randomly assigned in 6-well plates, and each test consisted of three replicates of 30 larvae per well. Zebrafish embryos were observed for mortality after 5 days of continuous exposure to different concentrations of compounds (5, 10, 20, 40, 80 μM). The control group was administered 0.01% DMSO (*v*/*v*). All of the test materials were diluted using E3 water, and the incubation solution was changed to a fresh one every day. All animal experiments were conducted in compliance with the requirements of the Animal Care and Use Committee of Northwest A&F University (No. XN2023-0706).

### 4.7. Behavioral Observation of Zebrafish Larvae

Behavioral experiments were conducted to assess the ameliorative effects of compounds on MPTP-induced PD-like dyskinesia in zebrafish larvae [49]. Two dpf zebrafish embryos were randomly assigned in 24-well plates, with 15 per well. Zebrafish embryos were co-cultured with different concentrations of compounds and MPTP (final concentration of 50 μM) for 5 consecutive days, and the incubation solution was changed to a fresh one every day. Levodopa methyl ester hydrochloride (1 mM) was the positive agent. At 7 dpf, 8 larvae from each group were randomly selected into 6-well plate wells and washed three times with water before adding 3 mL of E3 water to each well. After a 10 min acclimatization period, movements of zebrafish larvae were recorded over a 30 min period, using an automated computerized video tracking system in a silent environment. Distance traveled and the average speed of zebrafish larvae in the tracking video were analyzed using Ethovision XT (Noldus, Holland).

### 4.8. Detection of ROS Generation in Zebrafish Larvae

After 2 dpf, zebrafish embryos were randomly assigned to 24-well plates (n = 3, 10 per well). Embryos were continuously treated with compound **9** (20, 40, 80 μM) or a positive drug and MPTP (50 μM) until 5 dpf. The drug incubation solution was removed, 30 μM 2′,7′-Dichlorodihydrofluorescein diacetate (DCFH-DA) solution was added, and the plates were incubated for 1 h at 28 °C in the dark [32]. At the end of the incubation, zebrafish larvae were washed three times with E3 water and anesthetized by adding 0.02% tricaine. Each individual larvae was photographed using a fluorescence stereomicroscope (Nikon, Tokyo, Japan) under the same parameters, and the fluorescence of the larvae was quantified using Fiji. Since the fluorescence signals in the fluorescence pictures of the different larvae varied both in depth and in distribution, we used the Integrated Density (IntDen) values to characterize the ROS content.

### 4.9. Determination of SOD Activity of Zebrafish Larvae In Vivo

Zebrafish embryos at 2 dpf were randomly assigned to 6-well plates (n = 3, 30 per well), and embryos were continuously exposed to each drug up to 5 dpf according to the procedure of the ROS assay test. Then, the larvae were collected into EP tubes, rinsed three times with water, and frozen in liquid nitrogen immediately after removal of the excess liquid. Protein concentration and SOD activity of the larvae in each group were determined using the BCA Protein Concentration Assay Kit and SOD Activity Assay Kit (Solarbio, Beijing, China).

### 4.10. Statistical Analysis

All statistical data are presented as mean ± SEM. One-way ANOVA with Dunnett’s test was employed to assess the differences between the control and exposed groups. *p* < 0.05 indicates a significant difference between groups.

## 5. Conclusions

Five undescribed *nor*-polyprenylated acylphloroglucinols (norperforatums A–E, **1**–**5**) with a cyclohexanone core, as well as four known analogues (**6**–**9**), were isolated from the aerial parts of *H. perforatum*. Their structures were determined by analysis of the comprehensive spectroscopic data. The isolated compounds were evaluated for neuroprotective activities in the MPTP-induced zebrafish model. Compound **9** significantly alleviated the MPTP-induced locomotor impairments. Moreover, oxidative stress was also attenuated by compound **9** treatment. This work provides a novel insight for studying the neuroprotective effect of *nor*-prenylated acylphloroglucinols and broader potential applications in the improved PD symptoms for *H. perforatum*.

## Figures and Tables

**Table 1:** ^1^H NMR (400 MHz) data of compounds **1**–**5** (*δ*_H_ in ppm, *J* in Hz, in CDCl_3_).

No.	1	2	3	4	5
2	3.83, s	3.72, s	3.88, s	3.90, s	3.82, s
4	2.58, ddd (12.5, 8.6, 3.8)	1.74, m	1.79, m	1.80, m	1.69, m
5a	1.96, m	2.12, m	1.74, m	2.49, m	
5b	1.53, d (5.4)	1.16, m	1.55, m	1.29, m	
6	2.45, m	2.35, m	2.39, m		
8	2.50, m	2.45, m	2.36, m	2.37, m	2.57, m
9a	1.05, d (6.8)	1.03, d (6.8)	0.96, overlapped	1.62, m	1.02, d (6.6)
9b				1.25, m	
10	1.06, d (6.8)	1.05, d (6.8)	0.96, overlapped	1.06, d (6.7)	1.08, d (6.6)
11	1.02, s	1.02, s	0.96, s	0.96, s	0.96, s
12a	1.49, m	2.26, m	1.39, m	1.51, m	1.41, m
12b	1.26, m			1.41, m	
13a	2.07, m	5.61, overlapped	1.99, m	2.01, m	1.93, m
13b	1.71, m		1.74, m	1.67, m	1.68, m
14	4.93, t (6.5)	5.61, overlapped	4.92, overlapped	4.93, t (7.1)	4.91, t (6.7)
16a	1.63, s	1.30, s	1.61, s	1.63, s	1.65, s
16b					
17	1.56, s	1.30, s	1.56, s	1.55, s	1.52, s
18a	5.53, dd (15.6, 8.8)	2.17, m	2.04, m	2.11, m	2.39, m
18b		1.69, m	1.70, m		1.36, m
19a	5.74, d (15.6)	5.09, overlapped	5.00, overlapped	5.13, overlapped	2.08, m
19b					1.67, m
20					5.12, t (7.8)
21	1.32, s	1.71, s	1.67, s	1.74, s	
22	1.32, s	1.59, s	1.61, s	1.59, s	1.73, s
23a	2.39, m	2.37, m	2.32, m	2.51, m	1.61, s
23b	1.95, m	1.92, m	2.21, m	2.26, dd (14.5, 7.6)	
24a	5.07, t (6.5)	5.06, overlapped	4.96, overlapped	5.05, overlapped	2.45, m
24b					2.28, m
25					4.95, t (7.0)
26	1.68, s	1.68, s	1.65, s	1.68, s	
27	1.60, s	1.59, s	1.54, s	1.55, s	1.73, s
28				0.88, t (7.4)	1.56, s
29a					2.52, m
29b					2.33, m
30a				3.76, OCH_3_	2.28, m
30b					2.20, m
31					5.02, t (7.3)
33					1.61, s
34					1.52, s

**Table 2:** 13C NMR (100 MHz) data of compounds 1–5 (δC in ppm, in CDCl3).

No.	1	2	3	4	5
1	209.7, C	210.4, C	212.4, C	205.4, C	207.9, C
2	66.7, CH	67.2, CH	64.2, CH	66.1, CH	66.5, CH
3	45.3, C	46.4, C	45.3, C	45.8, C	45.2, C
4	45.8, CH	42.8, CH	38.0, CH	40.0, CH	40.2, CH
5	35.7, CH_2_	34.8, CH_2_	31.3, CH_2_	36.4, CH_2_	211.2, C
6	51.0, CH	51.4, CH	49.8, CH	61.6, C	67.8, C
7	210.8, C	211.3, C	211.3, C	209.9, C	210.2, C
8	42.7, CH	42.7, CH	42.7, CH	49.2, CH	42.5, CH
9	18.9, CH_3_	19.0, CH_3_	18.4, CH_3_	25.8, CH_2_	18.8, CH_3_
10	17.8, CH_3_	17.9, CH_3_	17.7, CH_3_	14.5, CH_3_	17.7, CH_3_
11	17.4, CH_3_	16.8, CH_3_	17.6, CH_3_	17.2, CH_3_	17.5, CH_3_
12	37.6, CH_2_	39.1, CH_2_	37.3, CH_2_	36.6, CH_2_	36.7, CH_2_
13	21.9, CH_2_	121.4, CH	22.0, CH_2_	22.1, CH_2_	22.0, CH_2_
14	123.7, CH	142.2, CH	123.7, CH	123.7, CH	123.6, CH
15	131.7, C	70.9, C	131.7, C	131.9, C	131.8, C
16	25.8, CH_3_	30.2, CH_3_	25.7, CH_3_	25.9, CH_3_	25.8, CH_3_
17	17.9, CH_3_	30.2, CH_3_	17.7, CH_3_	17.8, CH_3_	17.8, CH_3_
18	125.6, CH	27.0, CH_2_	26.9, CH_2_	27.0, CH_2_	35.3, CH_2_
19	140.6, CH	123.0, CH	123.2, CH	122.7, CH	27.0, CH_2_
20	70.9, C	133.1, C	132.8, C	133.1, C	122.6, CH
21	30.2, CH_3_	26.0, CH_3_	25.8, CH_3_	26.1, CH_3_	133.4, C
22	29.9, CH_3_	18.1, CH_3_	18.0, CH_3_	18.2, CH_3_	26.0, CH_3_
23	27.6, CH_2_	27.7, CH_2_	30.6, CH_2_	33.4, CH_2_	18.1, CH_3_
24	121.4, CH	121.6, CH	121.0, CH	118.2, CH	33.0, CH_2_
25	133.7, C	133.4, C	133.9, C	135.5, C	118.4, CH
26	26.0, CH_3_	26.0, CH_3_	25.9, CH_3_	26.0, CH_3_	135.1, C
27	18.0, CH_3_	18.0, CH_3_	18.0, CH_3_	17.9, CH_3_	26.0, CH_3_
28				11.8, CH_3_	18.0, CH_3_
29				172.3, C	37.9, CH_2_
30				52.5, OCH_3_	22.2, CH_2_
31					122.6, CH
32					133.3, C
33					25.8, CH_3_
34					17.8, CH_3_

## Data Availability

Data are contained within the article and Supplementary Materials.

## Supplementary Materials

The following supporting information can be downloaded at: https://www.mdpi.com/article/10.3390/ijms26073096/s1.
