# Supplementary material for: Neuroprotective Effect of Nor-Prenylated Acylphloroglucinols from Hypericum perforatum L. (St John’s Wort) in the MPTP-Induced Zebrafish Model
Source: Int J Mol Sci. 2025 Mar 27;26(7):3096. doi: 10.3390/ijms26073096 (PMC11989108; doi:10.3390/ijms26073096)
Supplement: Supplementary file 1 [file ijms-26-03096-s001.zip › ijms-3491247-supplementary.pdf]

Supporting Information

Neuroprotective Effect of *Nor*-prenylated Acylphloroglucinols from *Hypericum perforatum* L. (St John's wort) in the MPTP-induced Zebrafish Model

Wuyang Liu†, Peng Zhao†, Yihan Liu, Xiangyan Meng, Jinyan Xie, Junmian Tian* and Jinming Gao*

Shaanxi Key Laboratory of Natural Products & Chemical Biology, College of Chemistry & Pharmacy, Northwest A&F University, Yangling 712100, Shaanxi, People's Republic of China

† These authors contributed equally to this work.

*Corresponding authors:

Email: tianjunmian@nwsuaf.edu.cn (J. Tian); jinminggao@nwsuaf.edu.cn (J. Gao).

Table of Contents

Spectra of physico-chemical properties of 1 4
Figure S1. ^{1}H NMR spectrum of **1** (400 MHz, $CDCl_3$)....................4
Figure S2. ^{13}C NMR spectrum of **1** (100 MHz, $CDCl_3$) 4
Figure S3. DEPT (θ = 135°) spectrum of **1** (100 MHz, $CDCl_3$)....................5
Figure S4. ^{1}H−^{1}H COSY spectrum of **1** (400 MHz, $CDCl_3$)....................5
Figure S5. HSQC spectrum of **1** (400 MHz, $CDCl_3$) 6
Figure S6. HMBC spectrum of **1** (400 MHz, $CDCl_3$) 6
Figure S7. NOESY spectrum of **1** (400 MHz, $CDCl_3$)....................7
Figure S8. HR-ESI-MS spectrum of **1** 7
Figure S9. Experimental CD spectrum (in MeOH) of **1** 8
Figure S10. Experimental UV spectrum of **1** 8
Figure S11. IR spectrum of **1** 9
Spectra of physico-chemical properties of 2 10
Figure S12. ^{1}H NMR spectrum of **2** (400 MHz, $CDCl_3$)....................10
Figure S13. ^{13}C NMR spectrum of **2** (100 MHz, $CDCl_3$) 10
Figure S14. DEPT (θ = 135°) spectrum of **2** (100 MHz, $CDCl_3$)....................11
Figure S15. ^{1}H−^{1}H COSY spectrum of **2** (400 MHz, $CDCl_3$)....................11
Figure S16. HSQC spectrum of **2** (400 MHz, $CDCl_3$) 12
Figure S17. HMBC spectrum of **2** (400 MHz, $CDCl_3$) 12
Figure S18. NOESY spectrum of **2** (400 MHz, $CDCl_3$)....................13
Figure S19. HR-ESI-MS spectrum of **2** 13
Figure S20. Experimental CD spectrum (in MeOH) of **2** 14
Figure S21. Experimental UV spectrum of **2** 14
Figure S22. IR spectrum of **2** 15
Spectra of physico-chemical properties of 3 16
Figure S23. ^{1}H NMR spectrum of **3** (400 MHz, $CDCl_3$)....................16
Figure S24. ^{13}C NMR spectrum of **3** (100 MHz, $CDCl_3$) 16
Figure S25. DEPT (θ = 135°) spectrum of **3** (100 MHz, $CDCl_3$)....................17
Figure S26. ^{1}H−^{1}H COSY spectrum of **3** (400 MHz, $CDCl_3$)....................17
Figure S27. HSQC spectrum of **3** (400 MHz, $CDCl_3$) 18
Figure S28. HMBC spectrum of **3** (400 MHz, $CDCl_3$) 18
Figure S29. NOESY spectrum of **3** (400 MHz, $CDCl_3$)....................19
Figure S30. HR-ESI-MS spectrum of **3** 19
Figure S31. Experimental CD spectrum (in MeOH) of **3** 20
Figure S32. Experimental UV spectrum of **3** 20
Figure S33. IR spectrum of **3** 21
Spectra of physico-chemical properties of 4 22
Figure S34. ^{1}H NMR spectrum of **4** (400 MHz, $CDCl_3$)....................22
Figure S35. ^{13}C NMR spectrum of **4** (100 MHz, $CDCl_3$) 22
Figure S36. DEPT (θ = 135°) spectrum of **4** (100 MHz, $CDCl_3$)....................23
Figure S37. ^{1}H−^{1}H COSY spectrum of **4** (400 MHz, $CDCl_3$)....................23
Figure S38. HSQC spectrum of **4** (400 MHz, $CDCl_3$) 24
Figure S39. HMBC spectrum of **4** (400 MHz, $CDCl_3$) 24

Figure S40. NOESY spectrum of **4** (400 MHz, $CDCl_3$)........25
Figure S41. HR-ESI-MS spectrum of **4** 25
Figure S42. Experimental CD spectrum (in MeOH) of **4** 26
Figure S43. Experimental UV spectrum of **4** 26
Figure S44. IR spectrum of **4** 27
Spectra of physico-chemical properties of 5 28
Figure S45. ^{1}H NMR spectrum of **5** (400 MHz, $CDCl_3$)........28
Figure S46. ^{13}C NMR spectrum of **5** (100 MHz, $CDCl_3$) 28
Figure S47. DEPT (θ = 135°) spectrum of **5** (100 MHz, $CDCl_3$)........29
Figure S48. ^{1}H−^{1}H COSY spectrum of **5** (400 MHz, $CDCl_3$)........29
Figure S49. HSQC spectrum of **5** (400 MHz, $CDCl_3$) 30
Figure S50. HMBC spectrum of **5** (400 MHz, $CDCl_3$) 30
Figure S51. NOESY spectrum of **5** (400 MHz, $CDCl_3$)........31
Figure S52. HR-ESI-MS spectrum of **5** 31
Figure S53. Experimental CD spectrum (in MeOH) of **5** 32
Figure S54. Experimental UV spectrum of **5** 32
Figure S55. IR spectrum of **5** 33
Figure S56. Results of toxicity tests of different compounds on zebrafish larvae 34
Figure S57. Effects of compound 9 on locomotor impairments induced by MPTP in three batches of zebrafish larvae. 34

Spectra of physico-chemical properties of 1

Figure S1. ^{1}H NMR spectrum of **1** (400 MHz, $CDCl_3$)

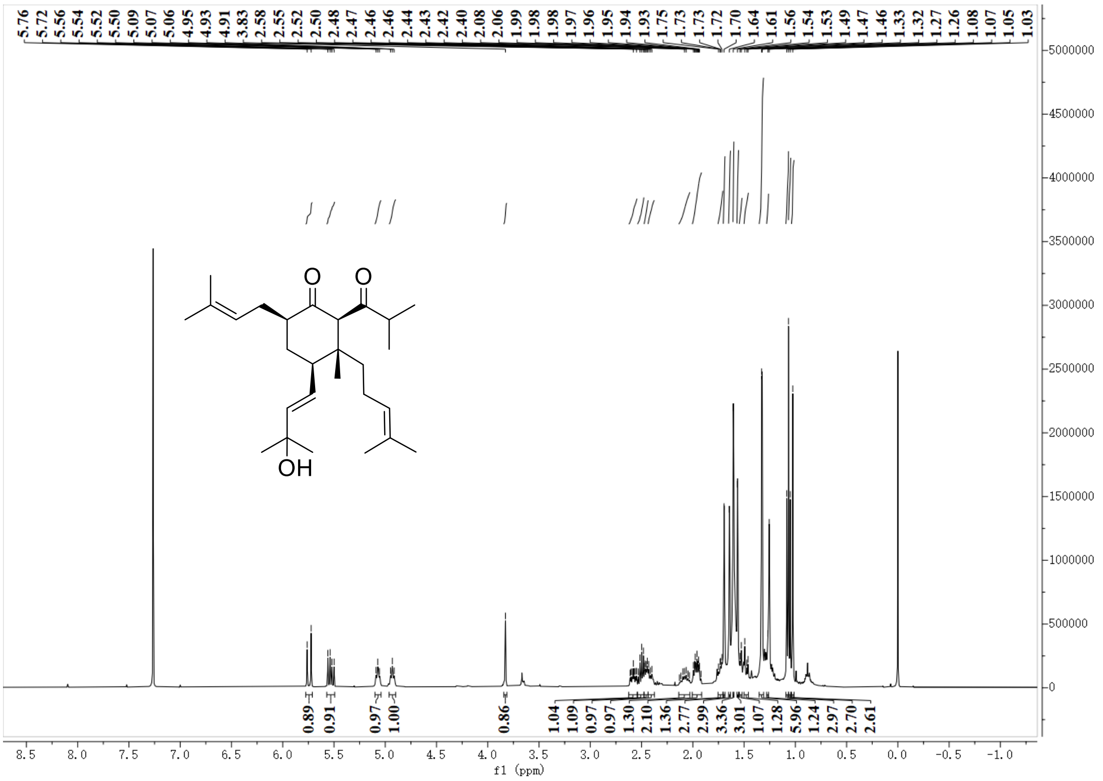

Figure S2. ^{13}C NMR spectrum of **1** (100 MHz, $CDCl_3$)

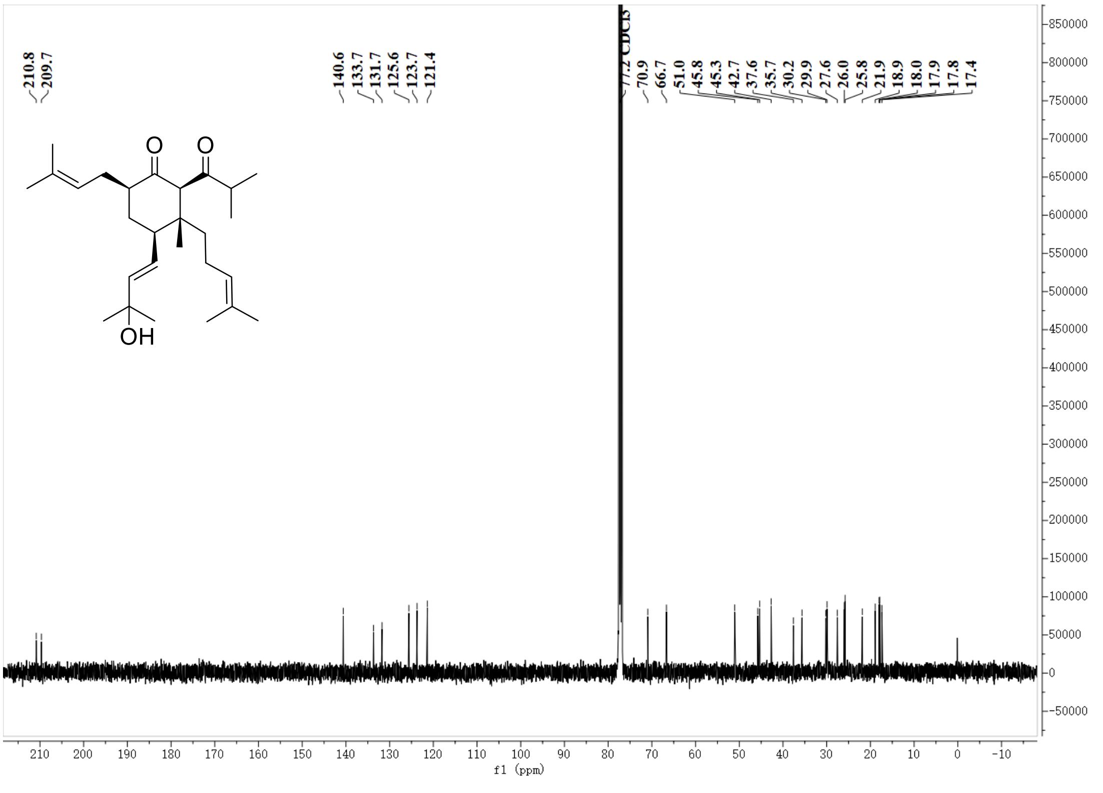

Figure S3. DEPT (θ = 135°) spectrum of **1** (100 MHz, $CDCl_3$)

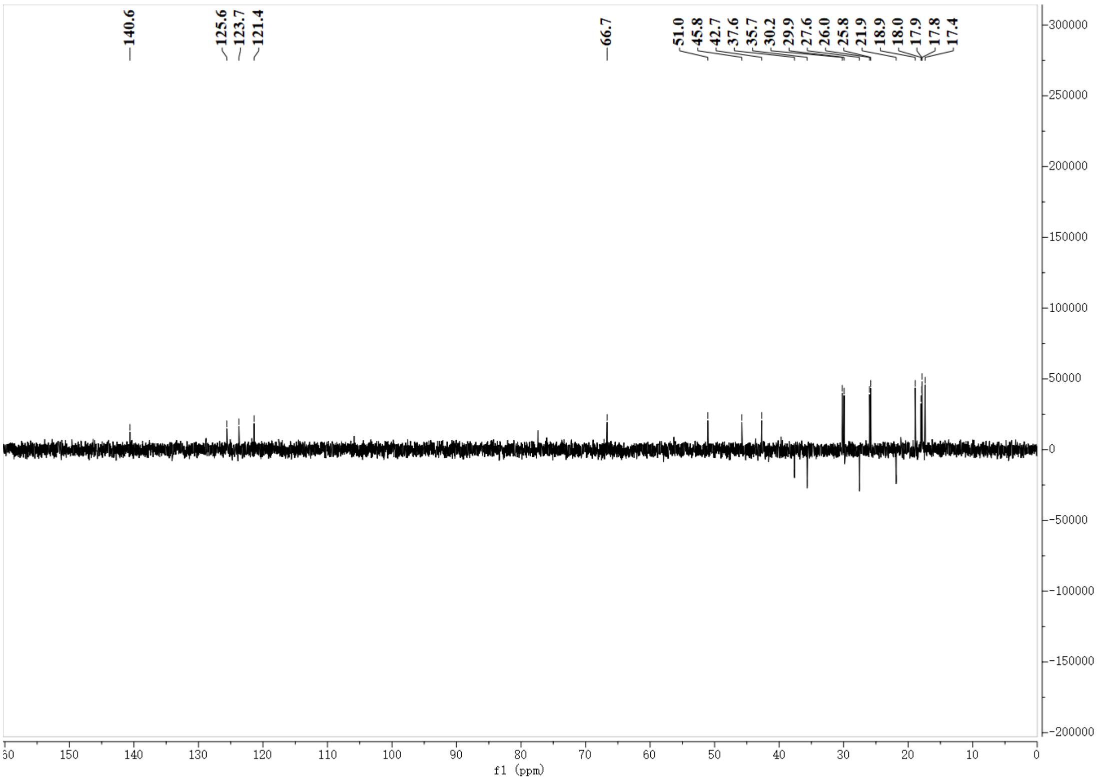

Figure S4. ^{1}H–^{1}H COSY spectrum of **1** (400 MHz, $CDCl_3$)

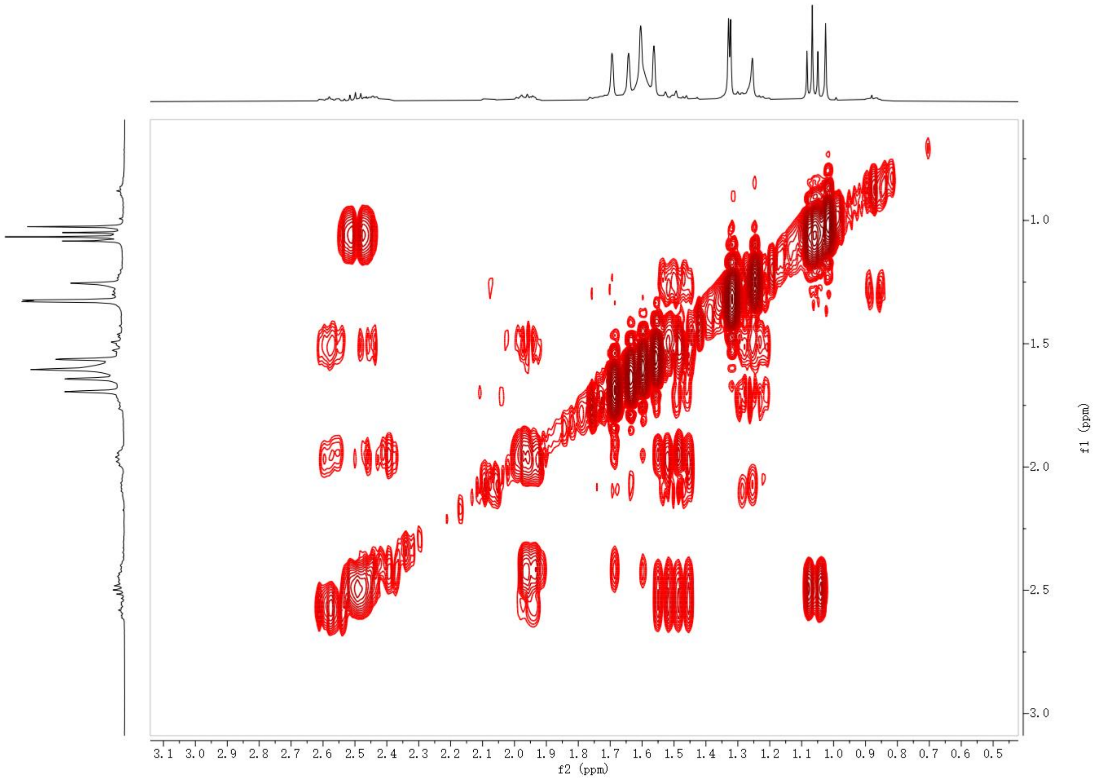

Figure S5. HSQC spectrum of **1** (400 MHz, $CDCl_3$)

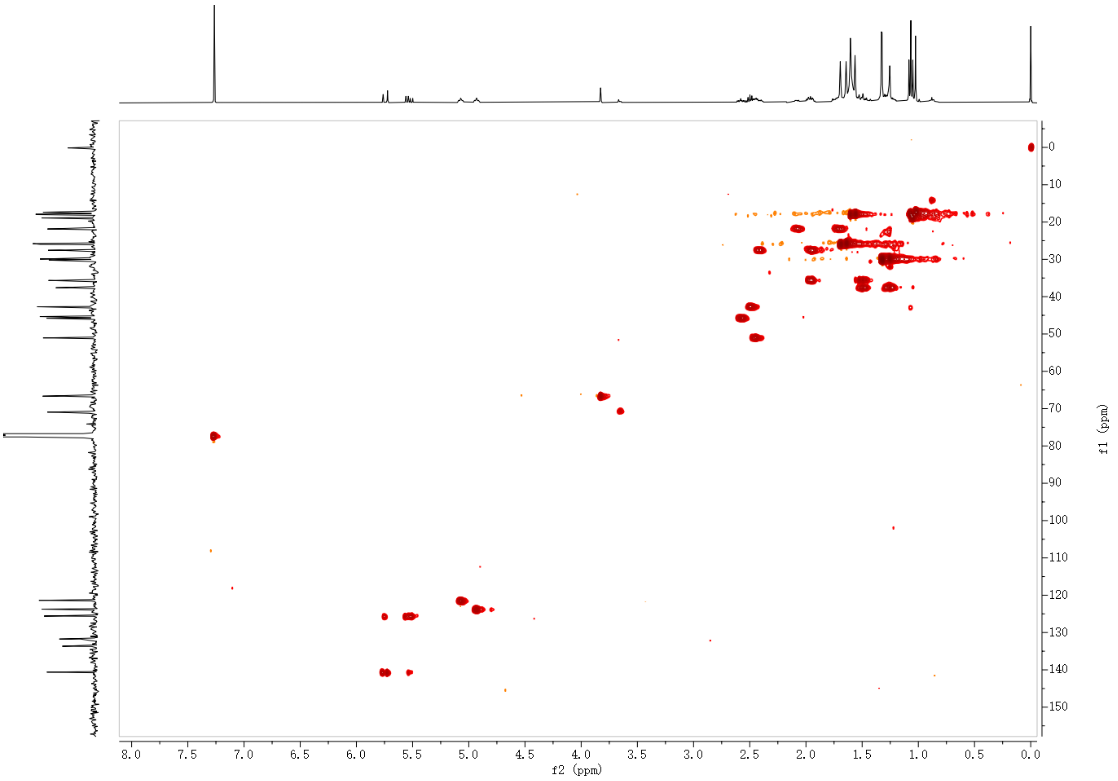

Figure S6. HMBC spectrum of **1** (400 MHz, $CDCl_3$)

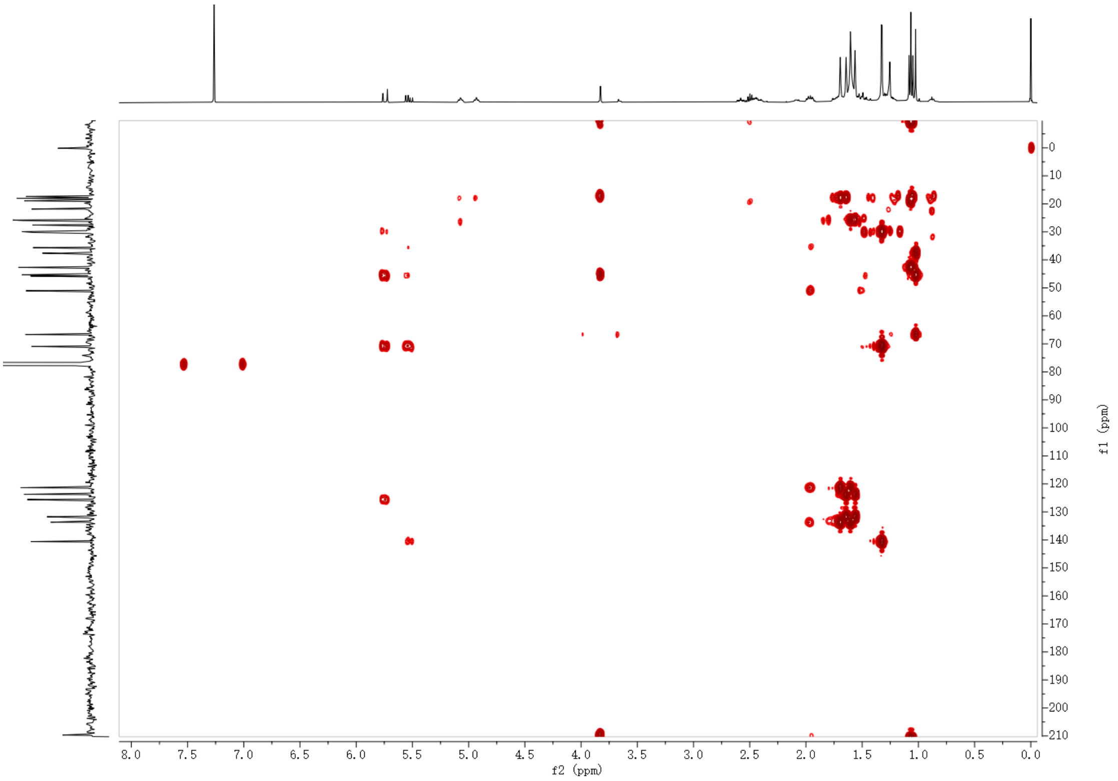

Figure S7. NOESY spectrum of **1** (400 MHz, $CDCl_3$)

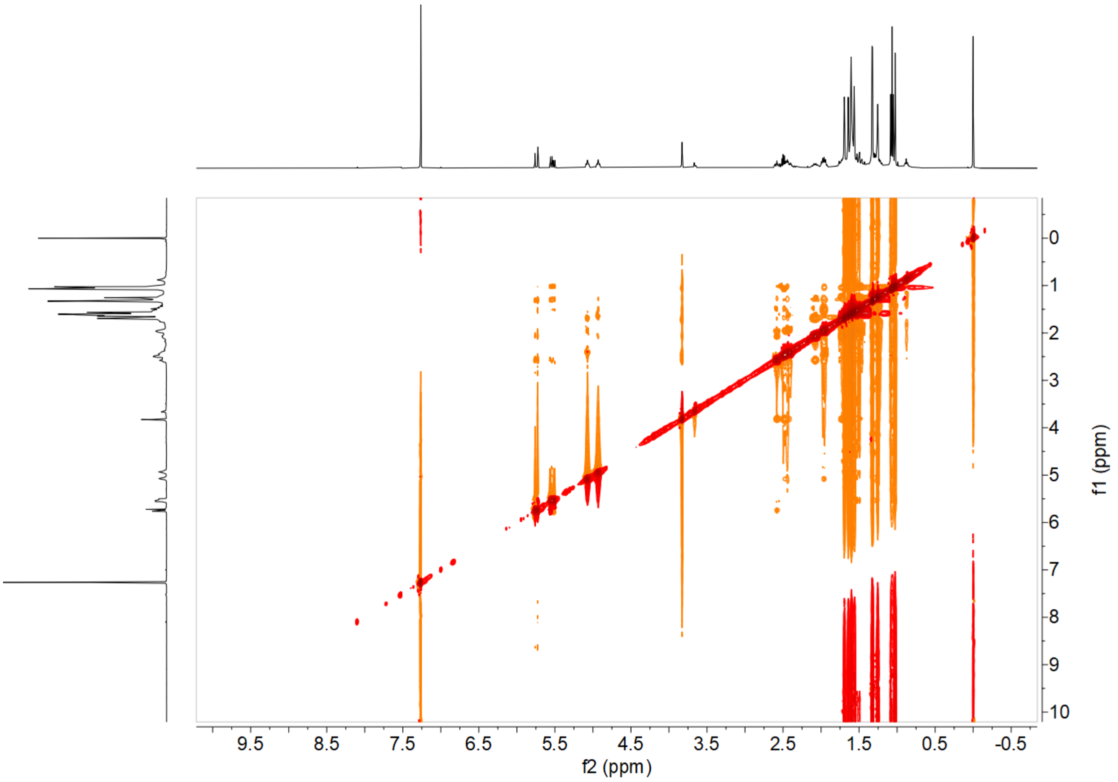

Figure S8. HR-ESI-MS spectrum of **1**

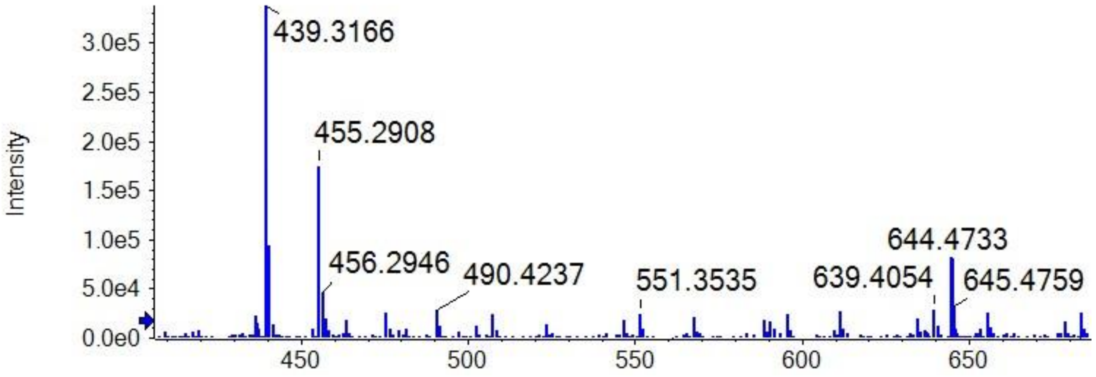

Figure S9. Experimental CD spectrum (in MeOH) of **1**

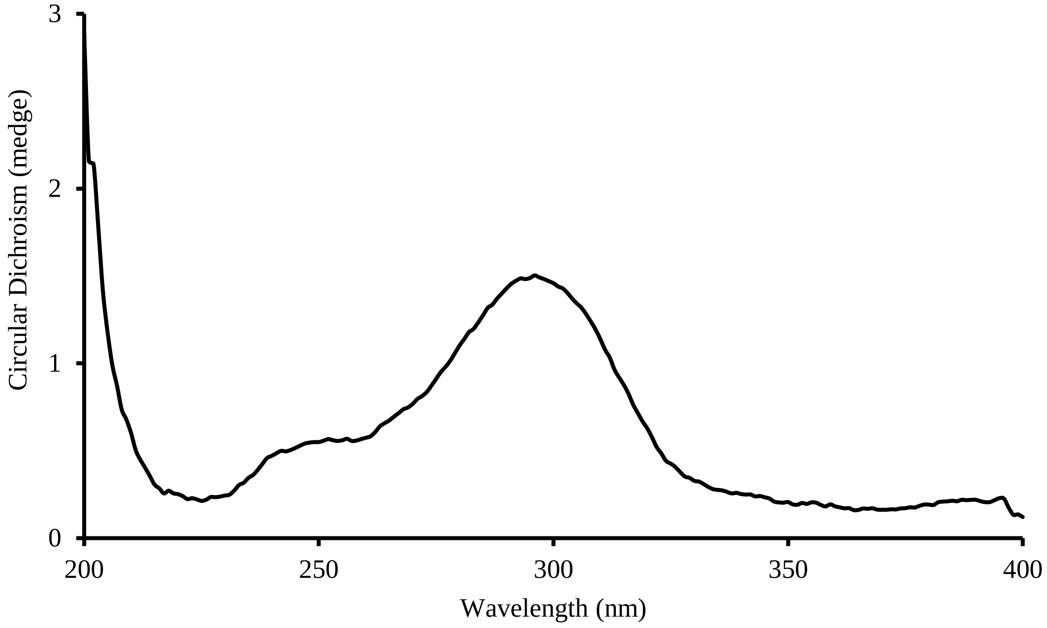

Figure S10. Experimental UV spectrum of **1**

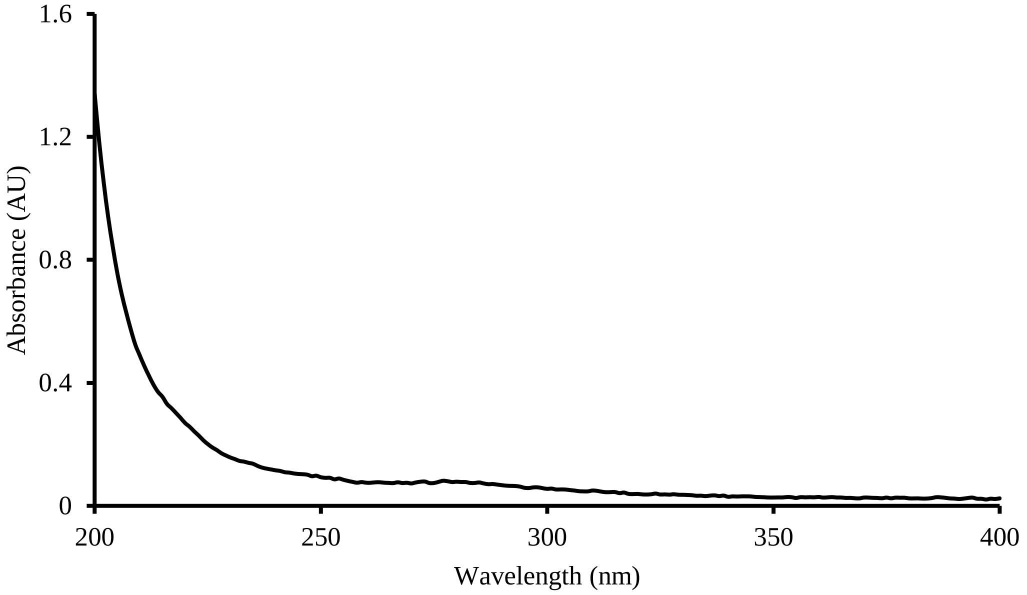

Figure S11. IR spectrum of **1**

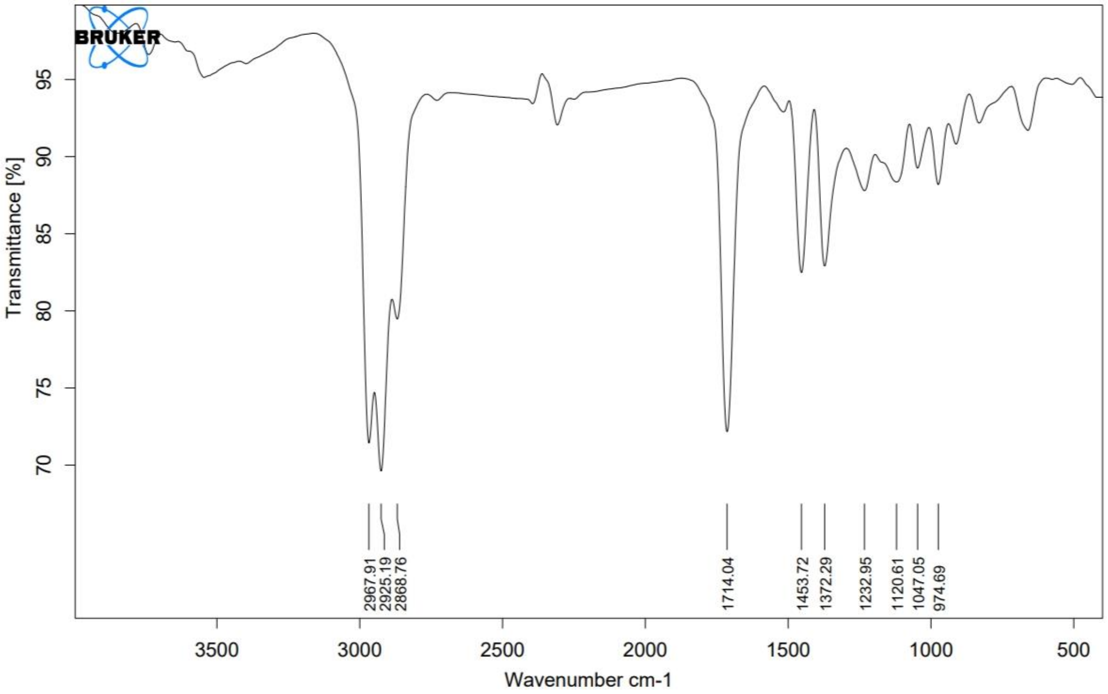

Spectra of physico-chemical properties of 2

Figure S12. ^{1}H NMR spectrum of **2** (400 MHz, $CDCl_3$)

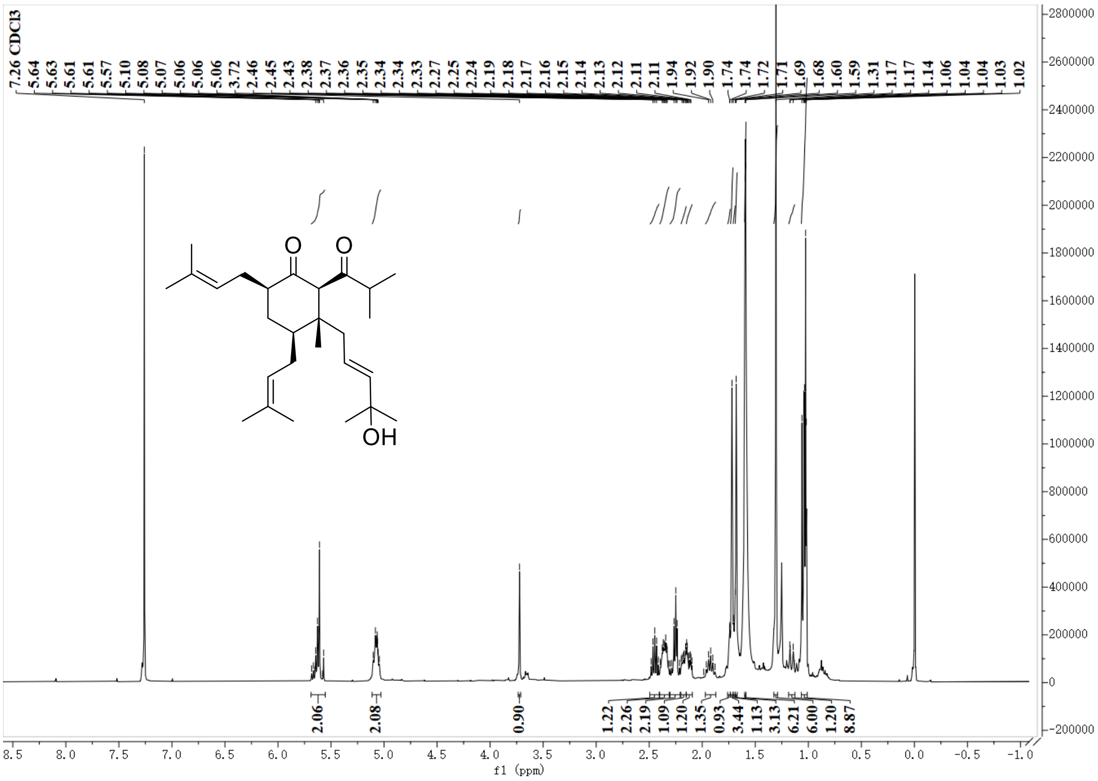

Figure S13. ^{13}C NMR spectrum of **2** (100 MHz, $CDCl_3$)

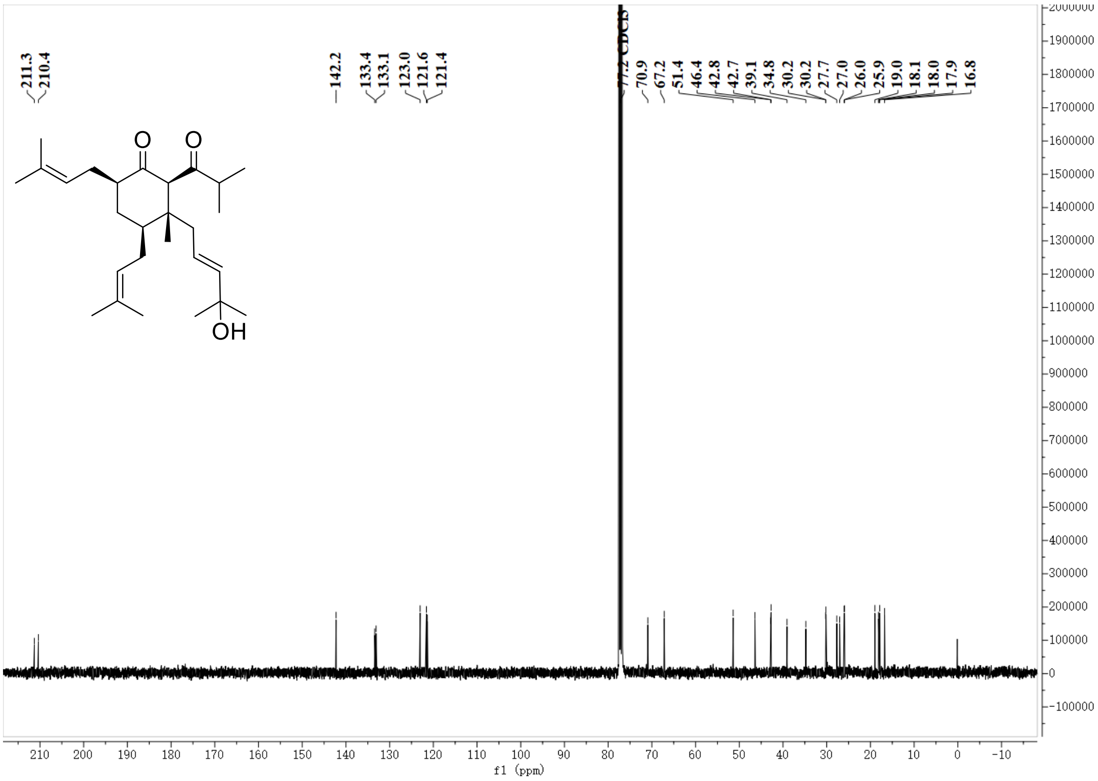

Figure S14. DEPT (θ = 135°) spectrum of **2** (100 MHz, $CDCl_3$)

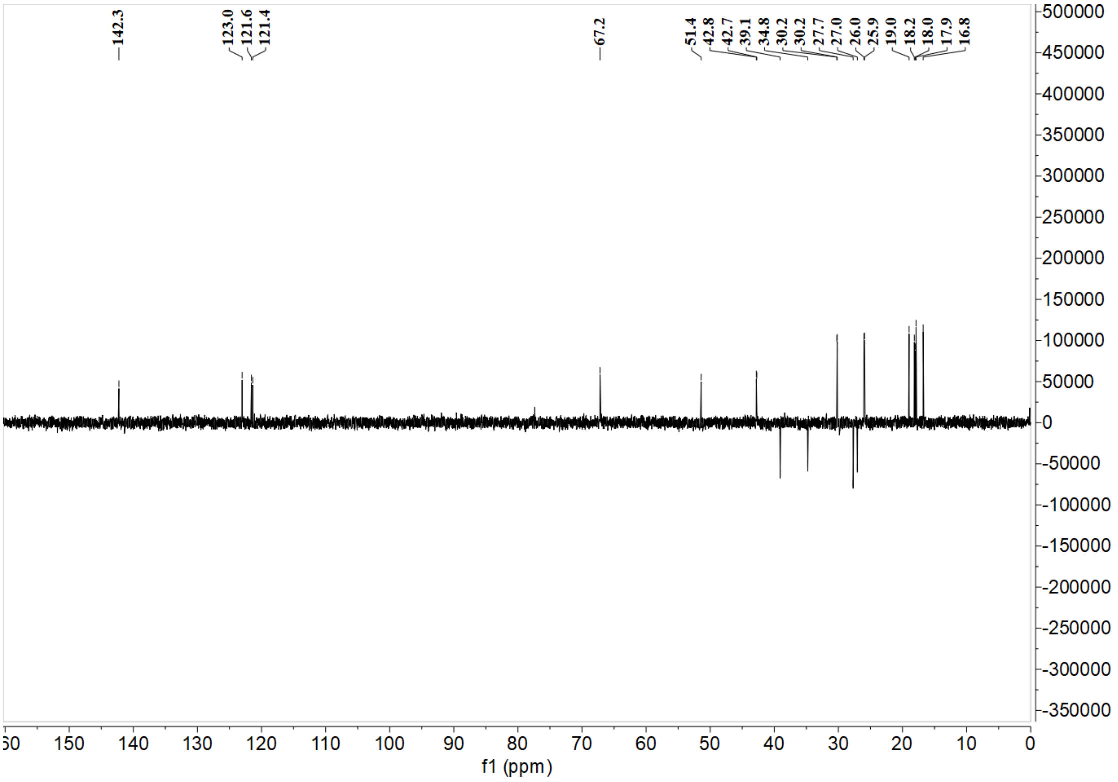

Figure S15. ^{1}H–^{1}H COSY spectrum of **2** (400 MHz, $CDCl_3$)

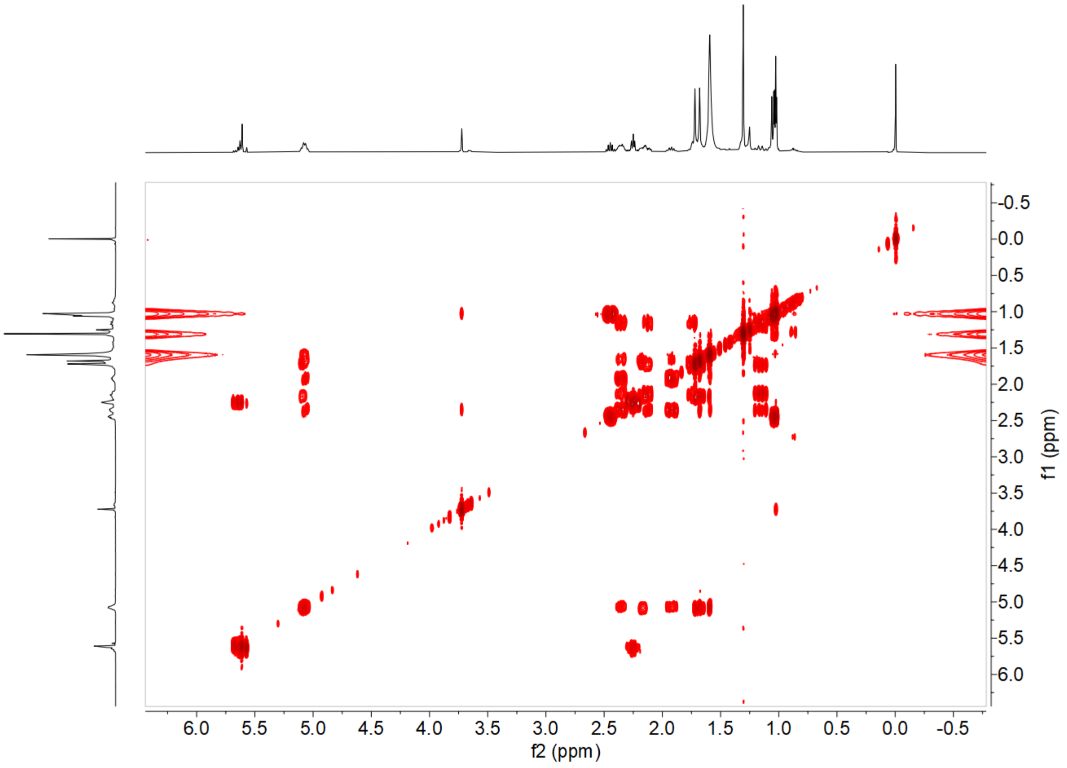

Figure S16. HSQC spectrum of **2** (400 MHz, $CDCl_3$)

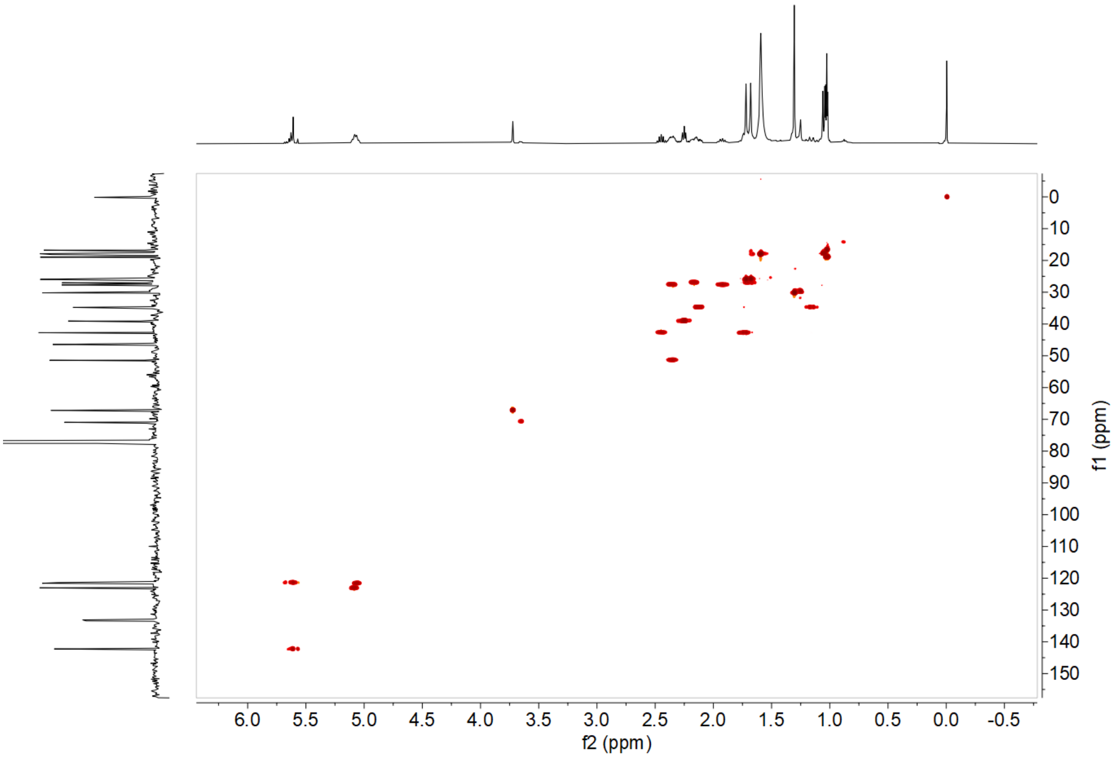

Figure S17. HMBC spectrum of **2** (400 MHz, $CDCl_3$)

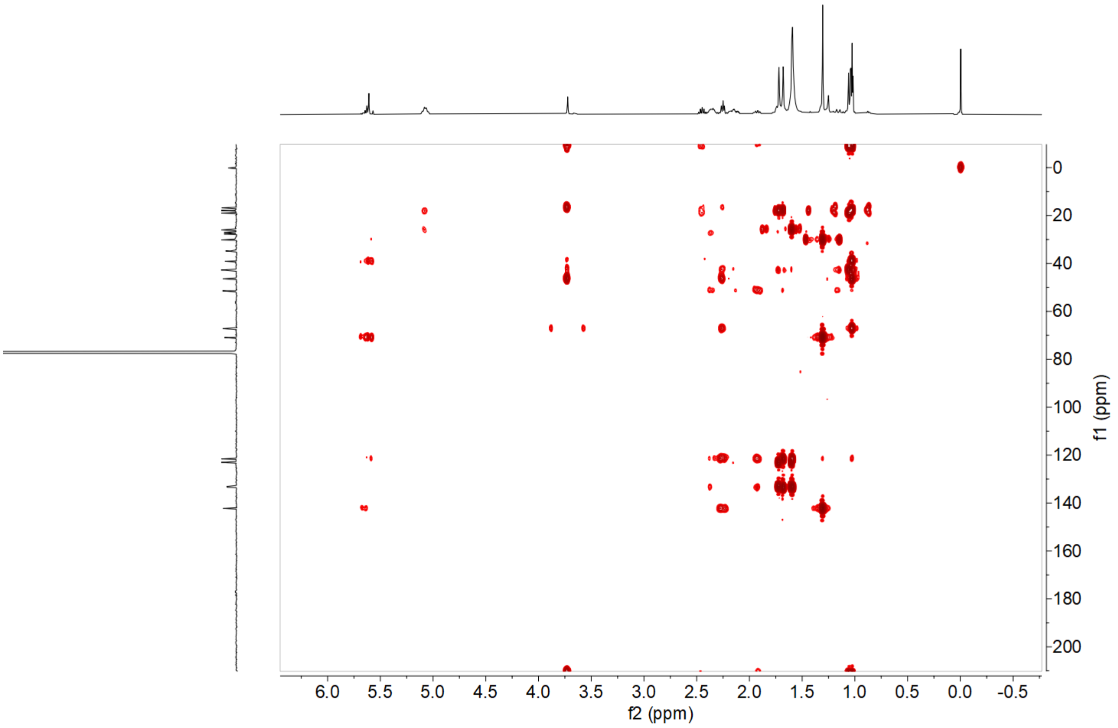

Figure S18. NOESY spectrum of **2** (400 MHz, $CDCl_3$)

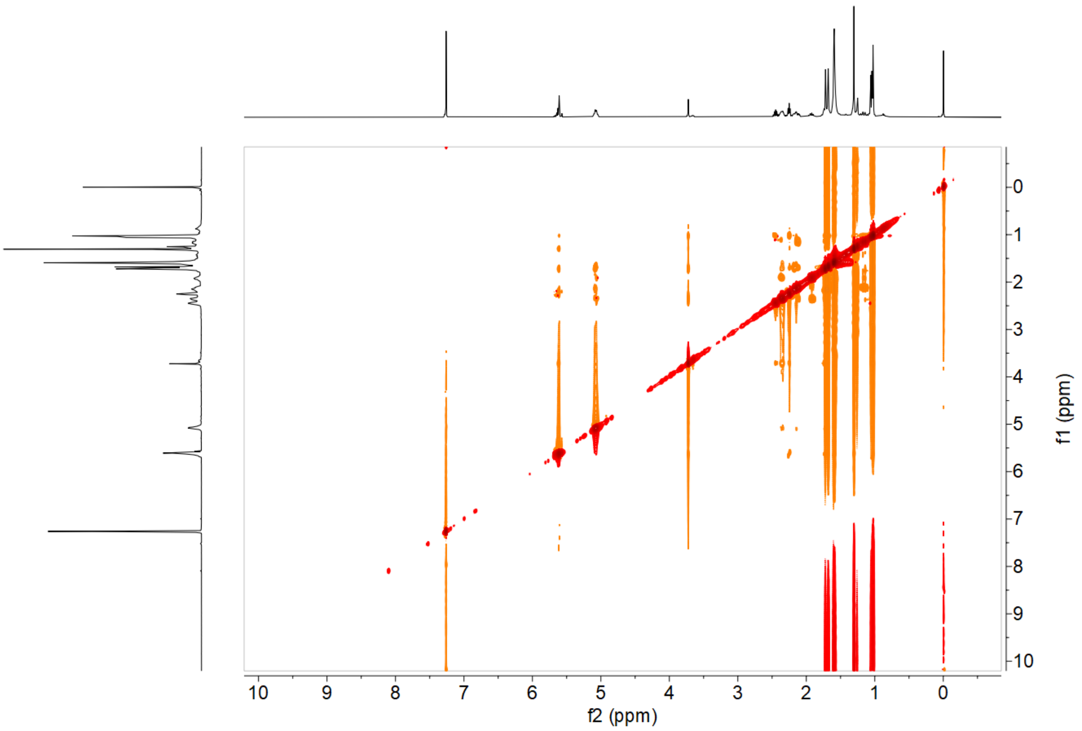

Figure S19. HR-ESI-MS spectrum of **2**

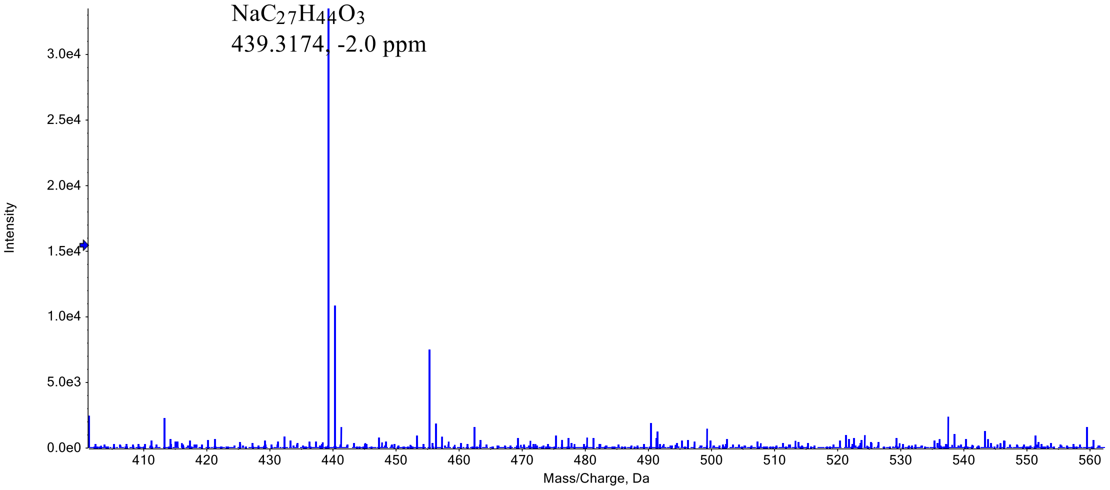

Figure S20. Experimental CD spectrum (in MeOH) of **2**

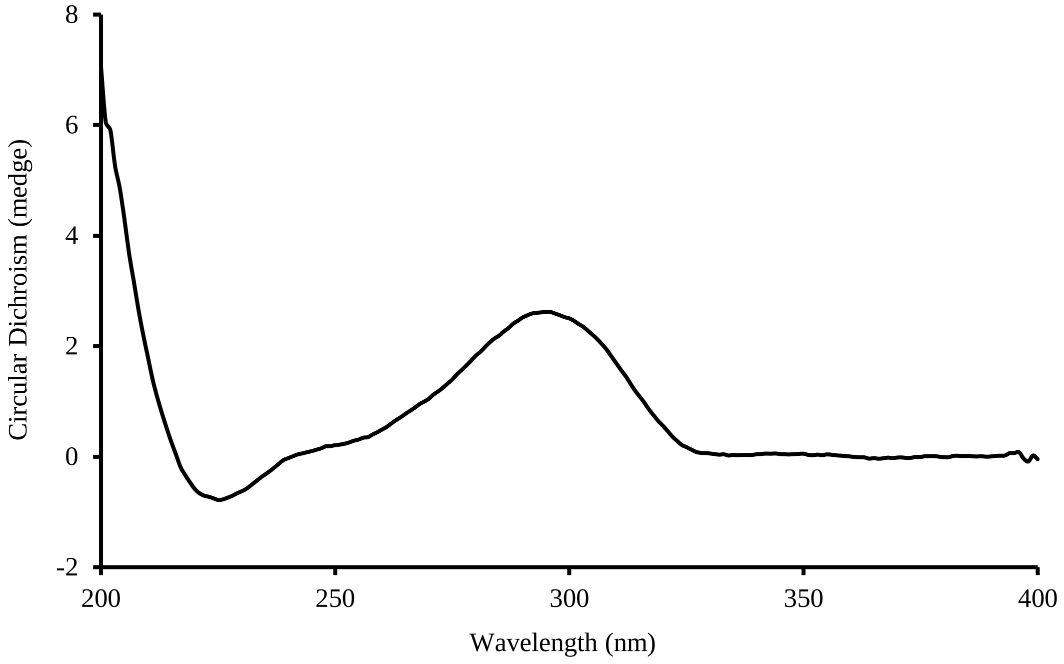

Figure S21. Experimental UV spectrum of **2**

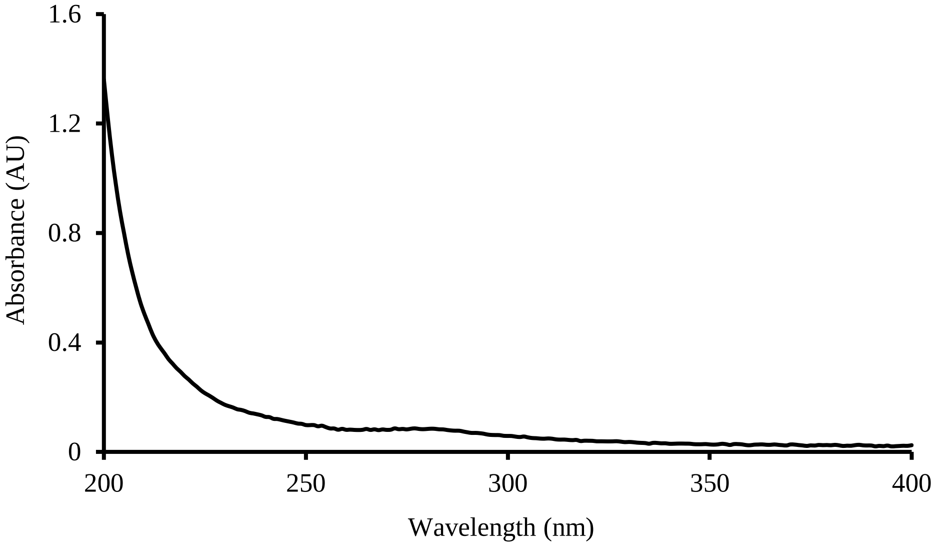

Figure S22. IR spectrum of **2**

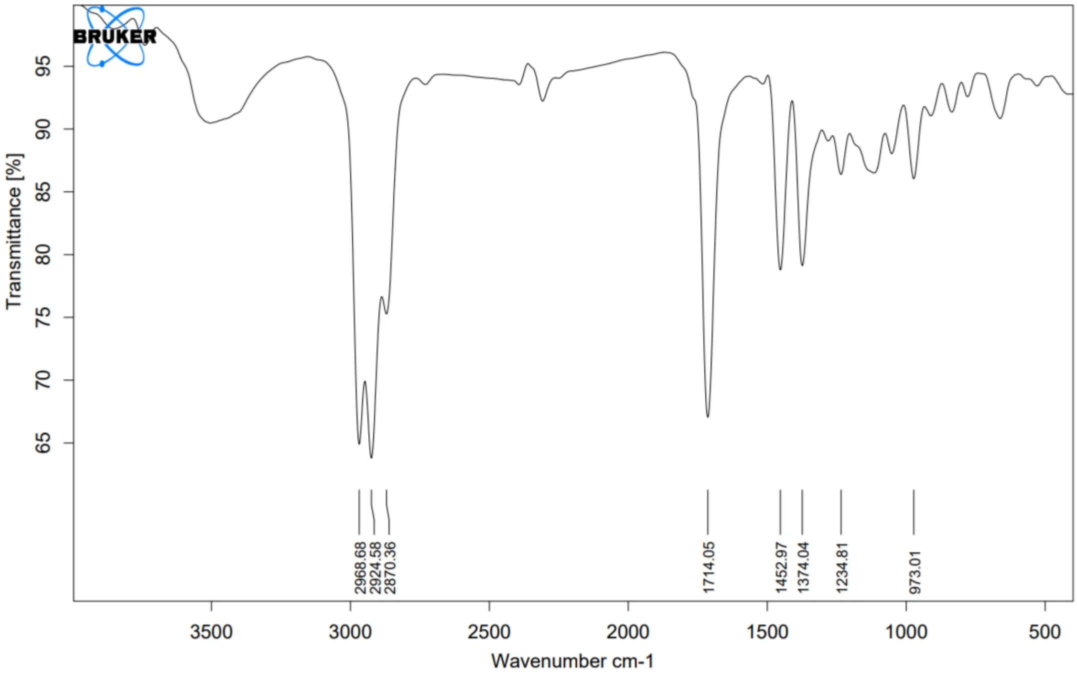

Spectra of physico-chemical properties of 3

Figure S23. ^{1}H NMR spectrum of **3** (400 MHz, $CDCl_3$)

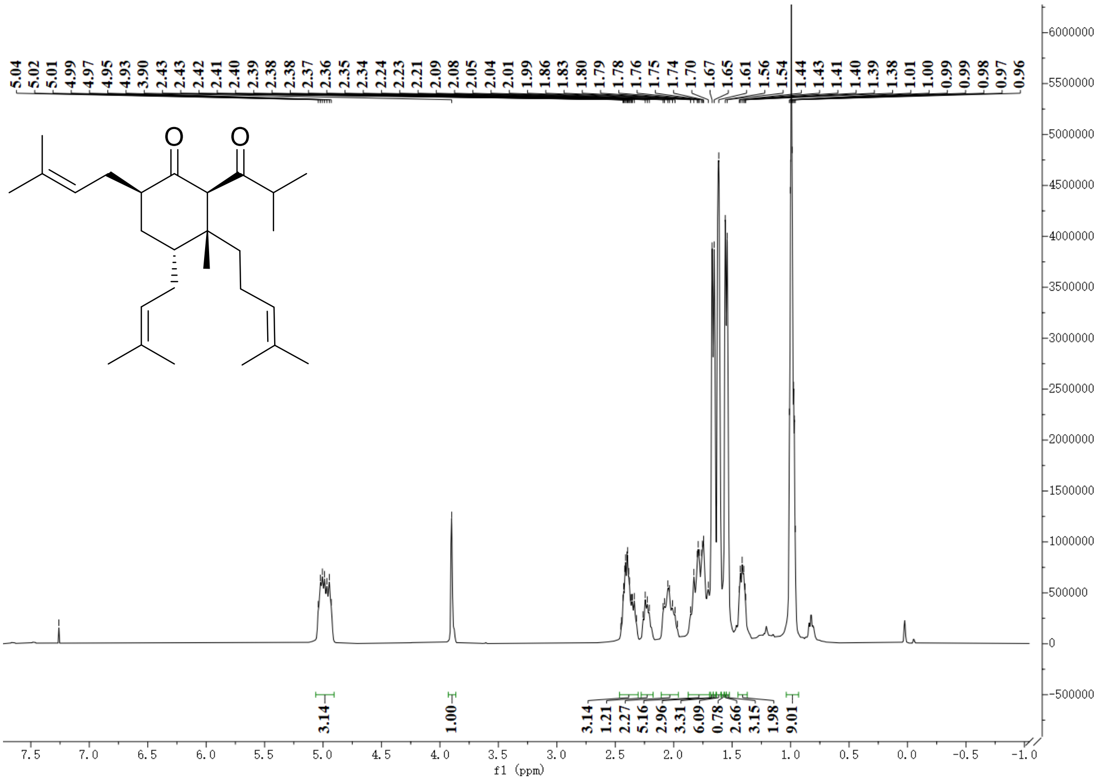

Figure S24. ^{13}C NMR spectrum of **3** (100 MHz, $CDCl_3$)

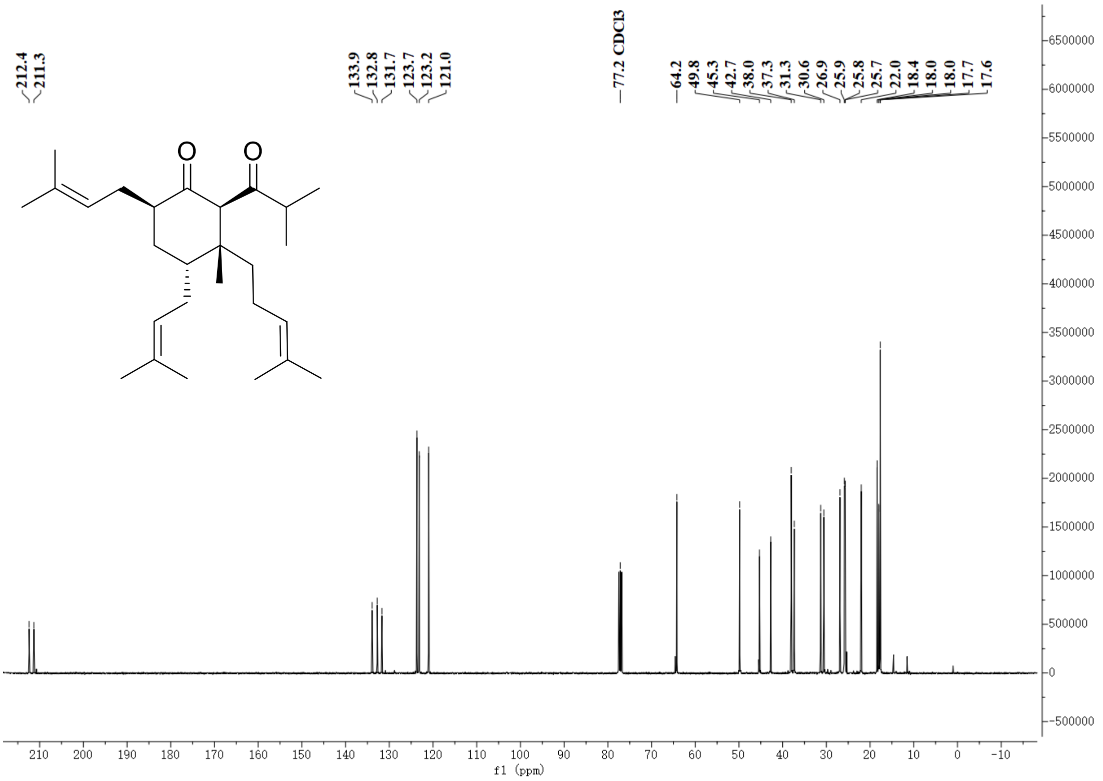

Figure S25. DEPT (θ = 135°) spectrum of **3** (100 MHz, $CDCl_3$)

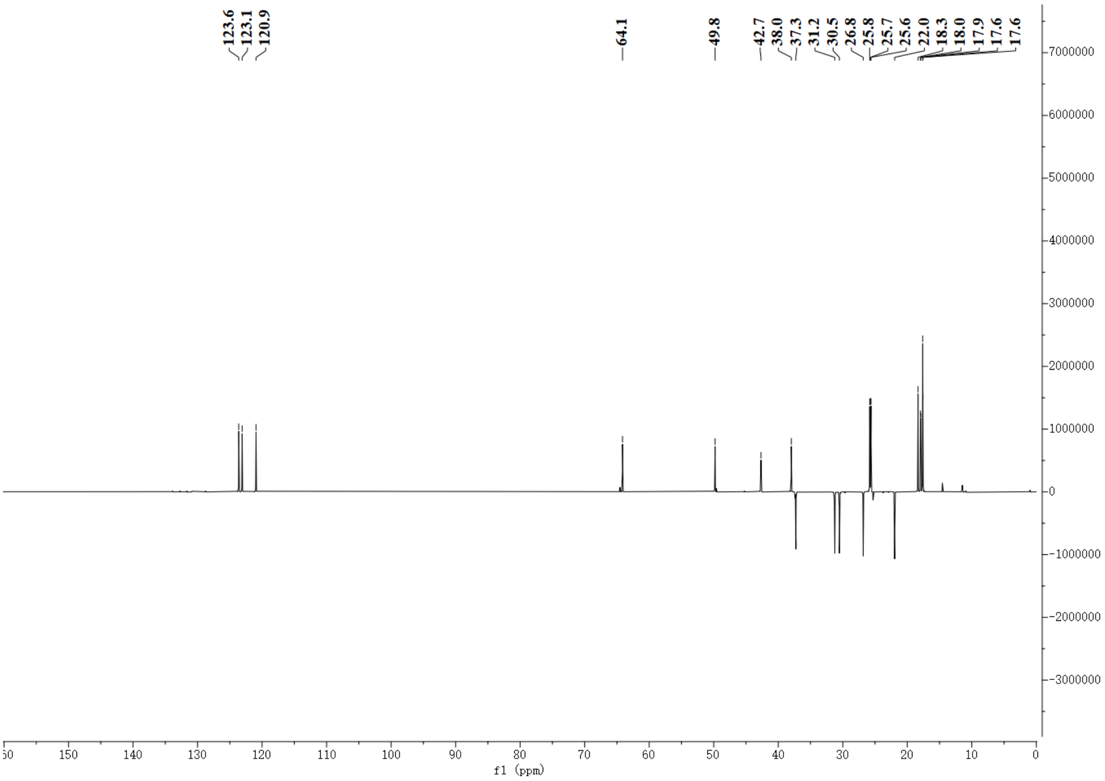

Figure S26. ^{1}H–^{1}H COSY spectrum of **3** (400 MHz, $CDCl_3$)

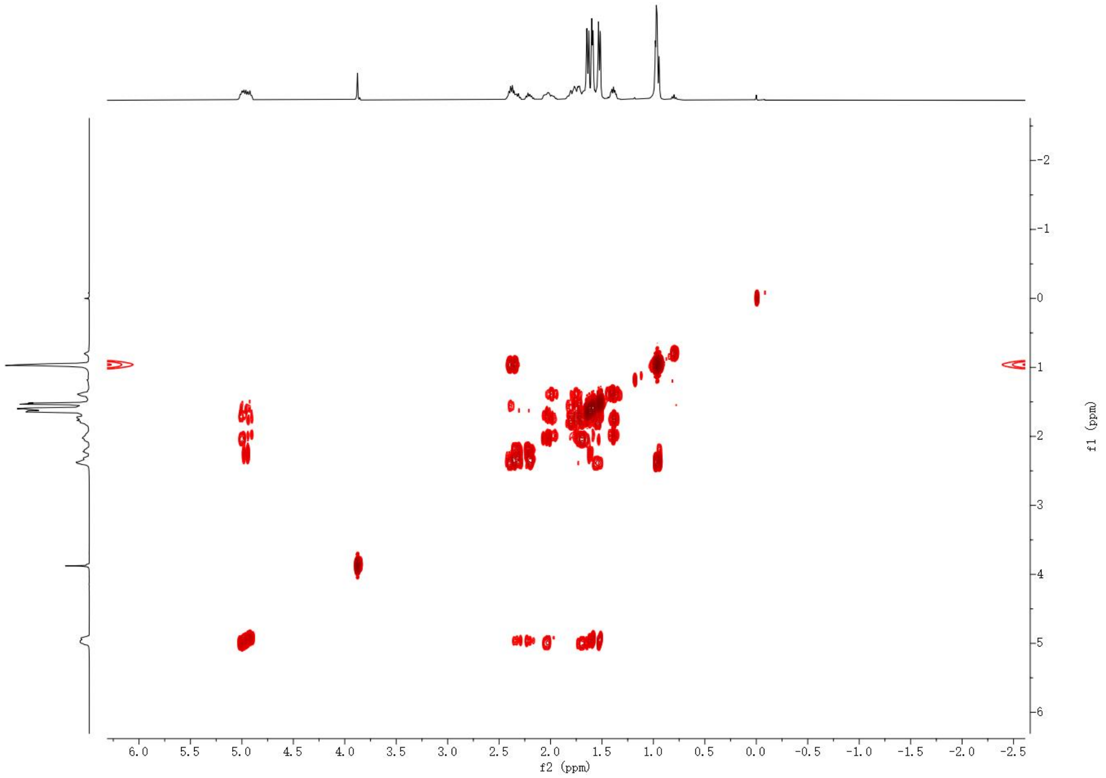

Figure S27. HSQC spectrum of **3** (400 MHz, $CDCl_3$)

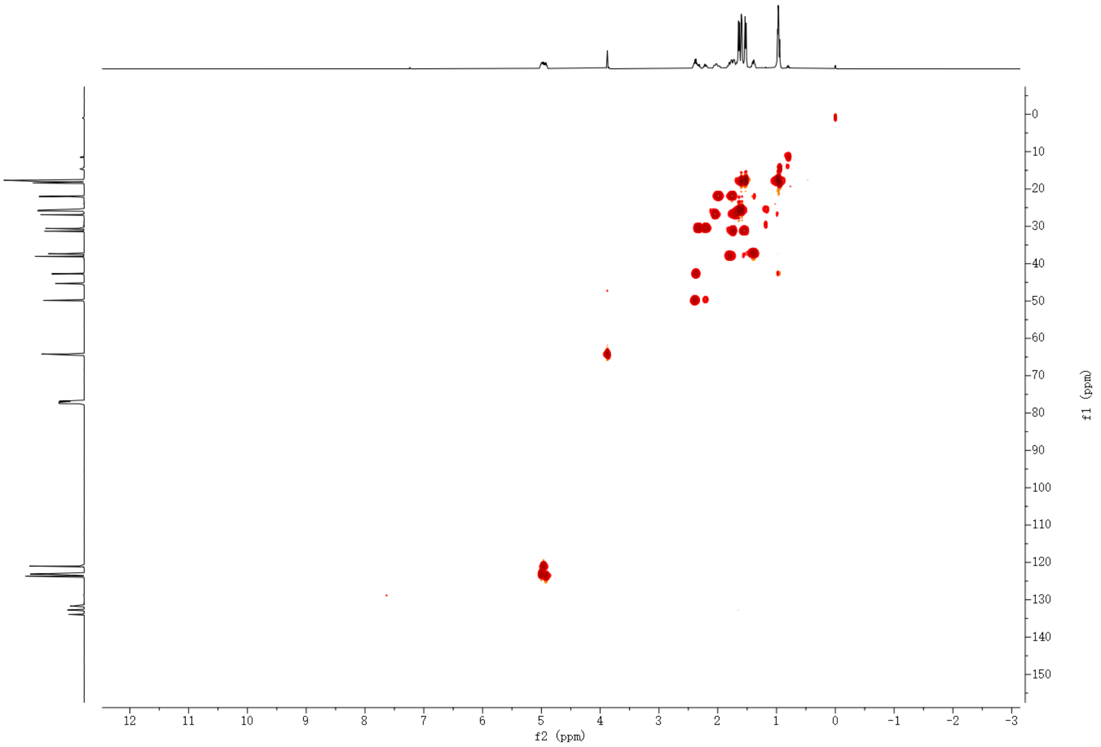

Figure S28. HMBC spectrum of **3** (400 MHz, $CDCl_3$)

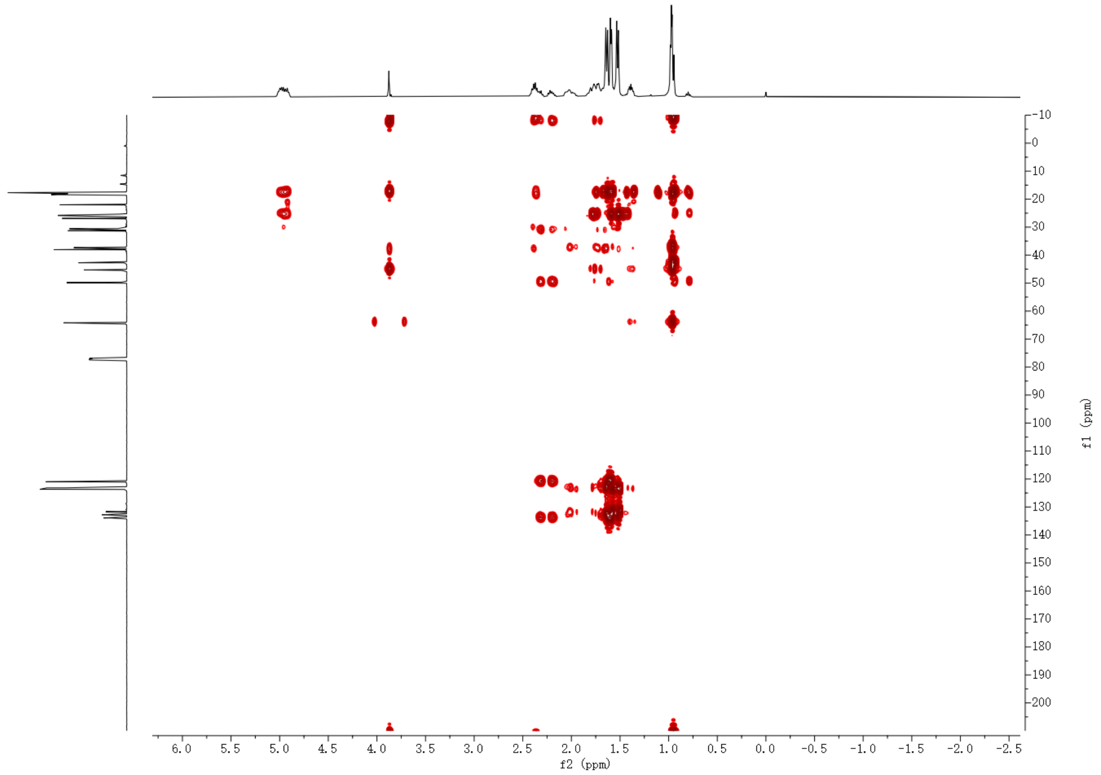

Figure S29. NOESY spectrum of **3** (400 MHz, $CDCl_3$)

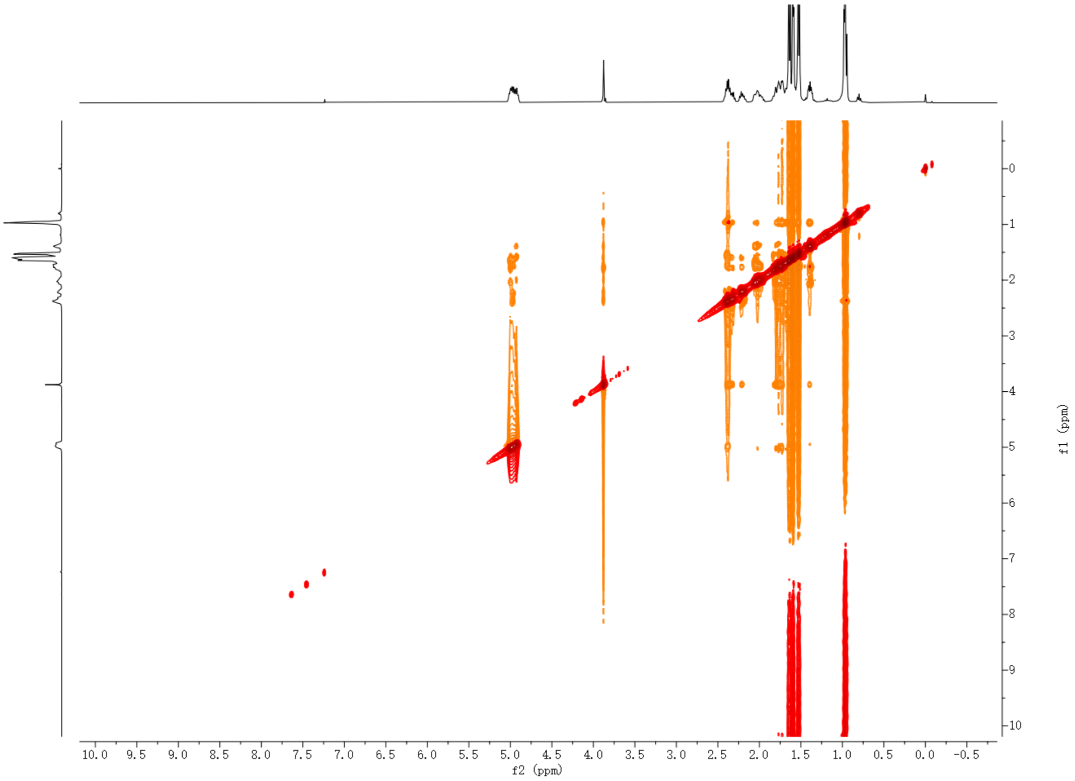

Figure S30. HR-ESI-MS spectrum of **3**

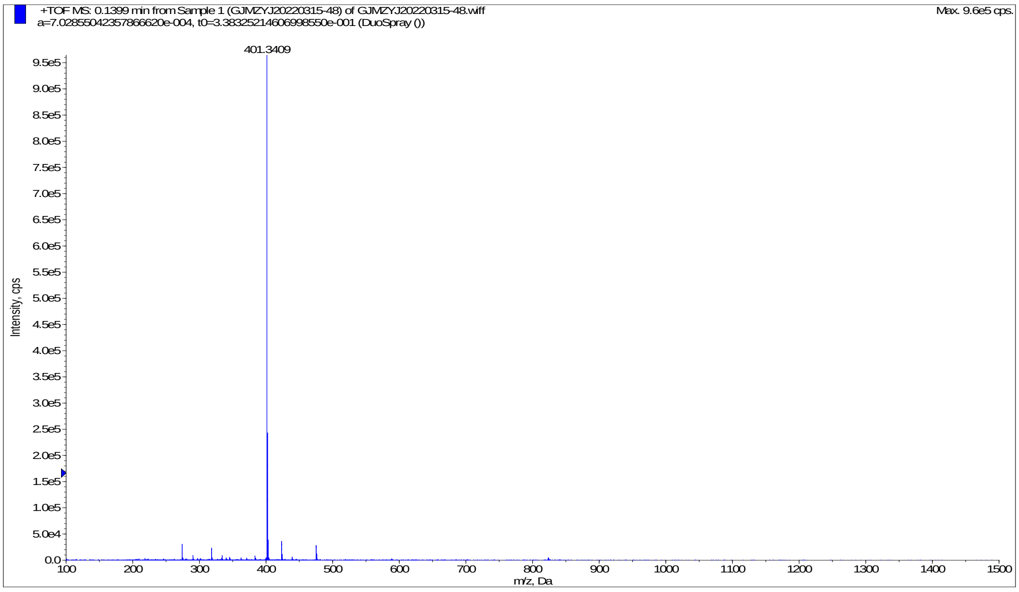

Figure S31. Experimental CD spectrum (in MeOH) of **3**

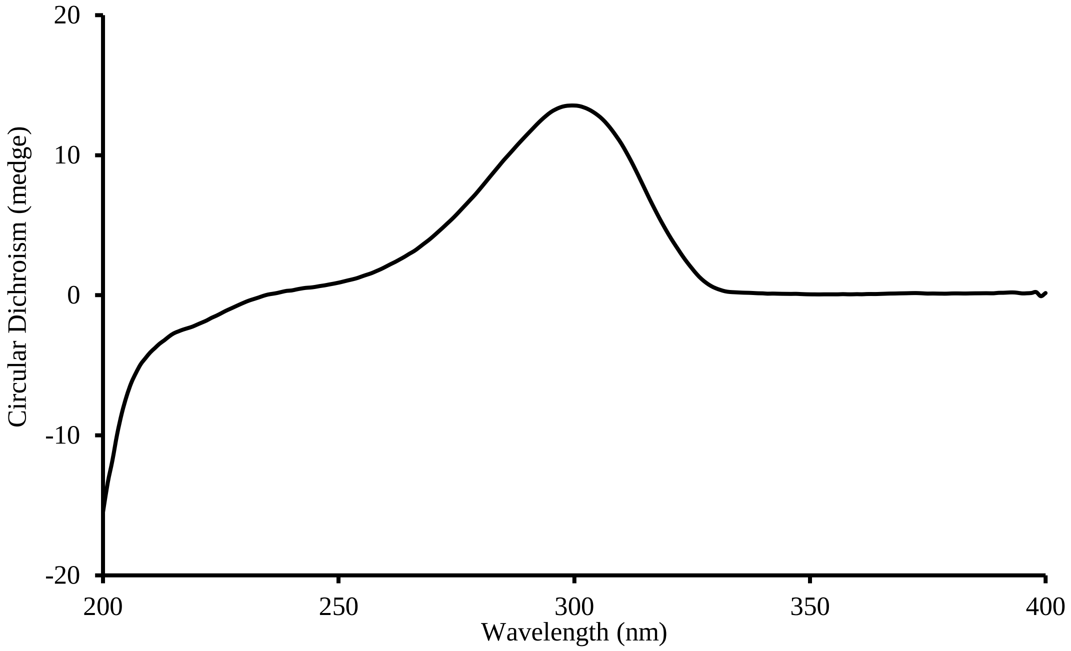

Figure S32. Experimental UV spectrum of **3**

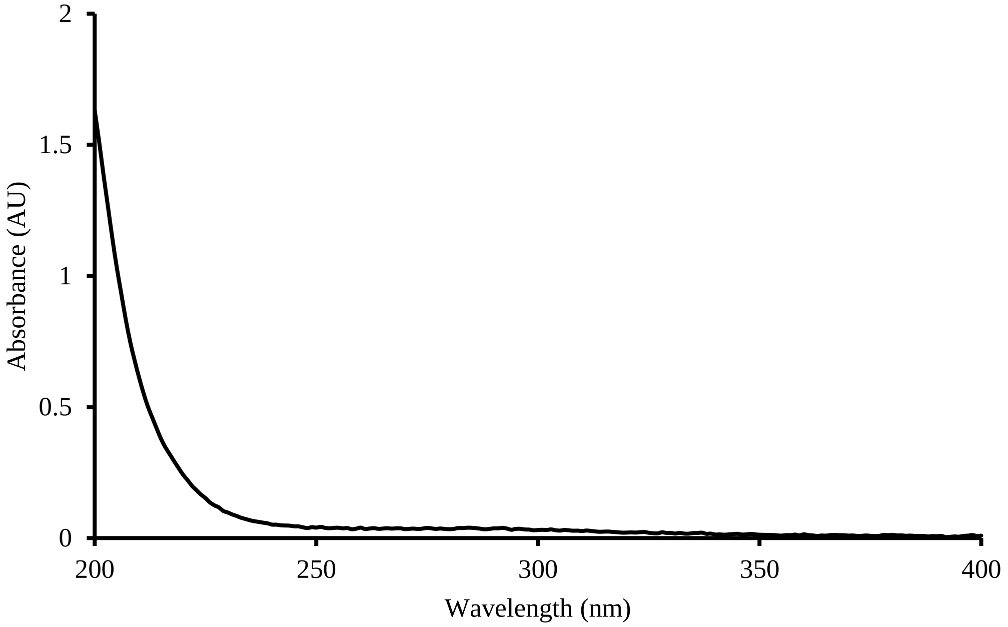

Figure S33. IR spectrum of **3**

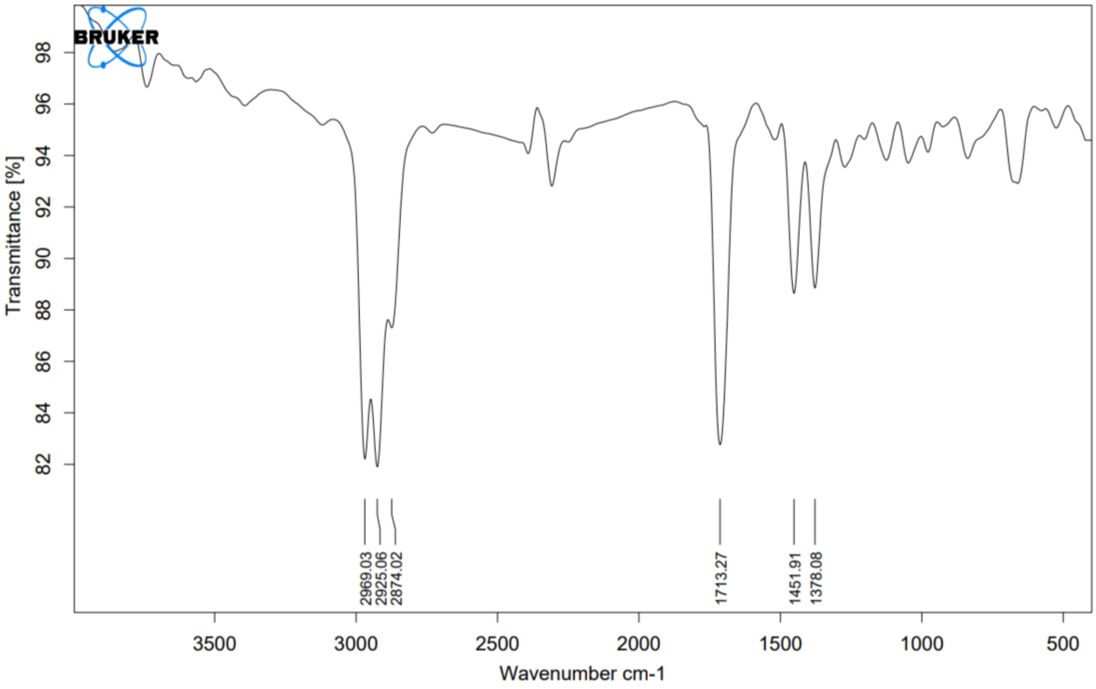

Spectra of physico-chemical properties of 4

Figure S34. ^{1}H NMR spectrum of **4** (400 MHz, $CDCl_3$)

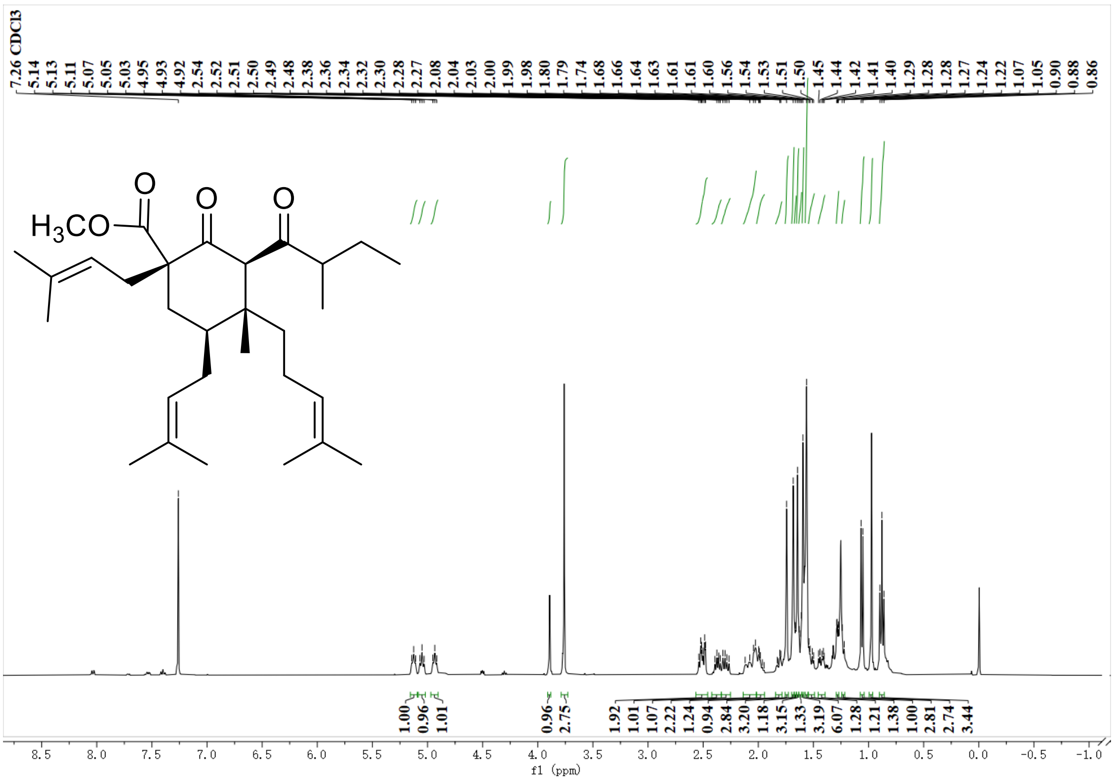

Figure S35. ^{13}C NMR spectrum of **4** (100 MHz, $CDCl_3$)

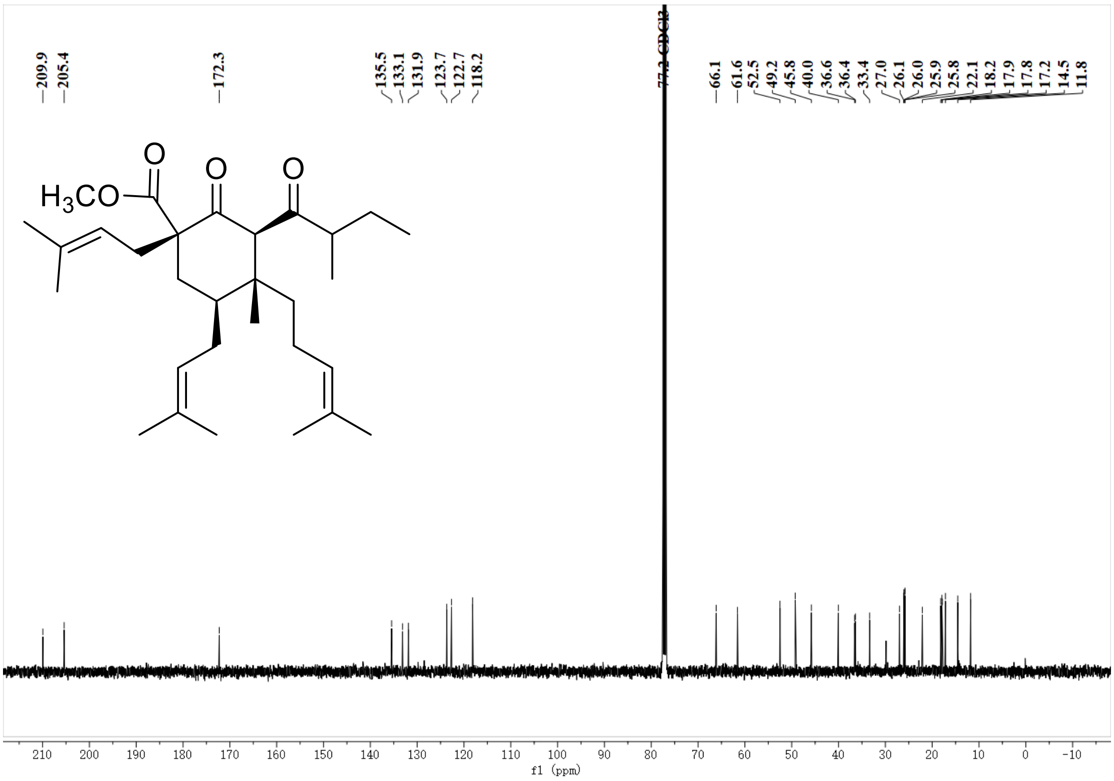

Figure S36. DEPT (θ = 135°) spectrum of **4** (100 MHz, $CDCl_3$)

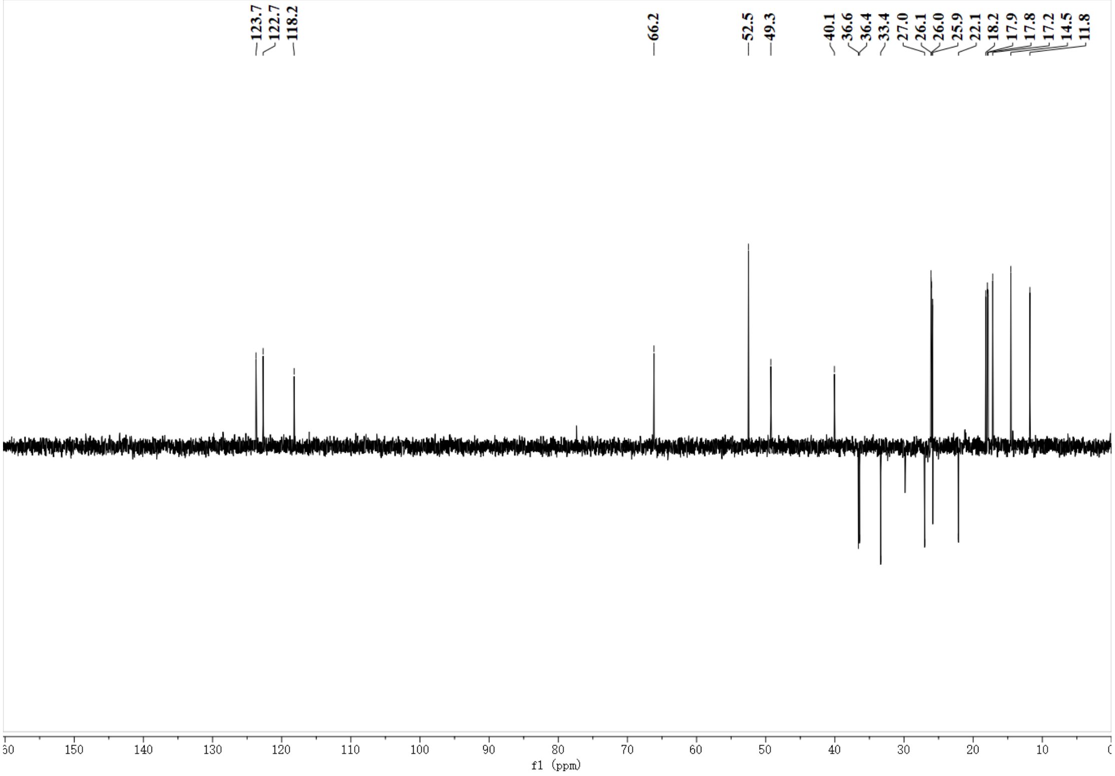

Figure S37. ^{1}H–^{1}H COSY spectrum of **4** (400 MHz, $CDCl_3$)

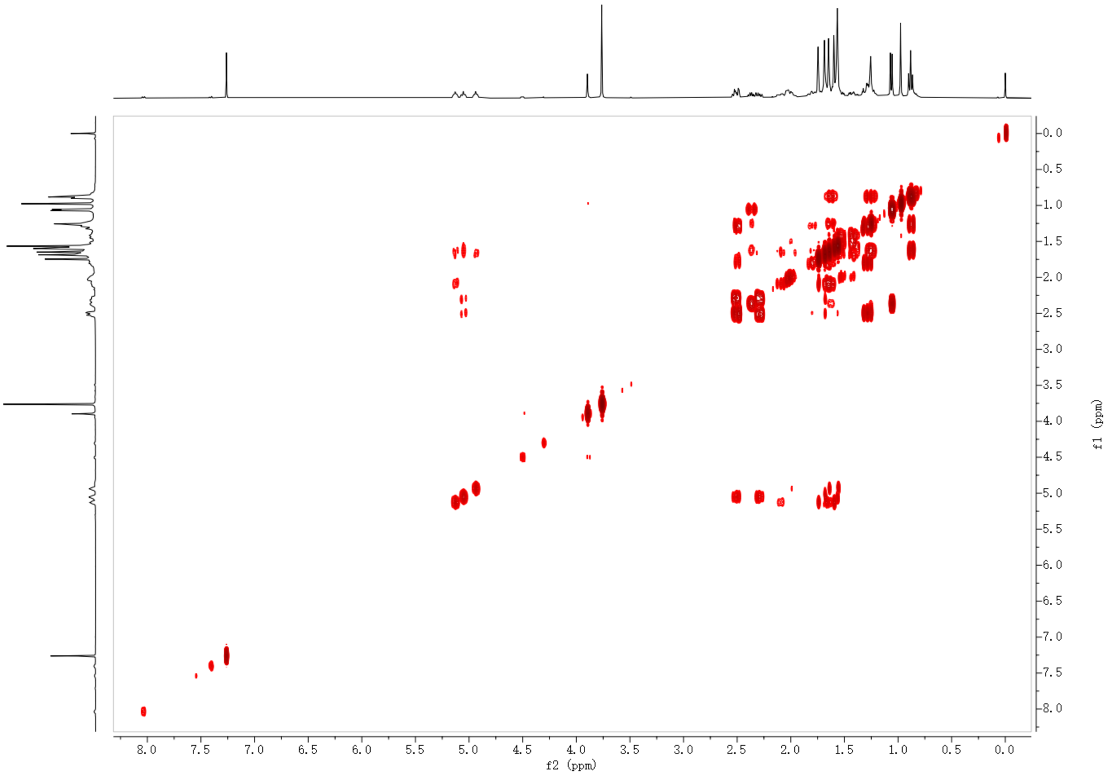

Figure S38. HSQC spectrum of **4** (400 MHz, $CDCl_3$)

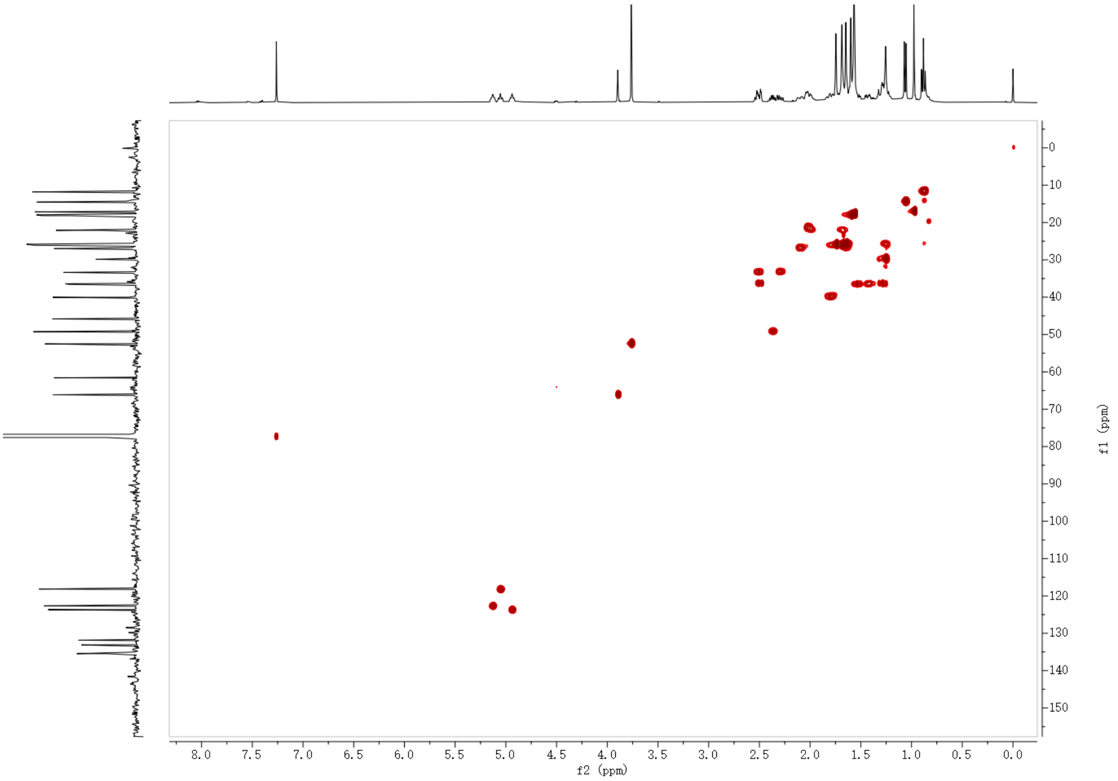

Figure S39. HMBC spectrum of **4** (400 MHz, $CDCl_3$)

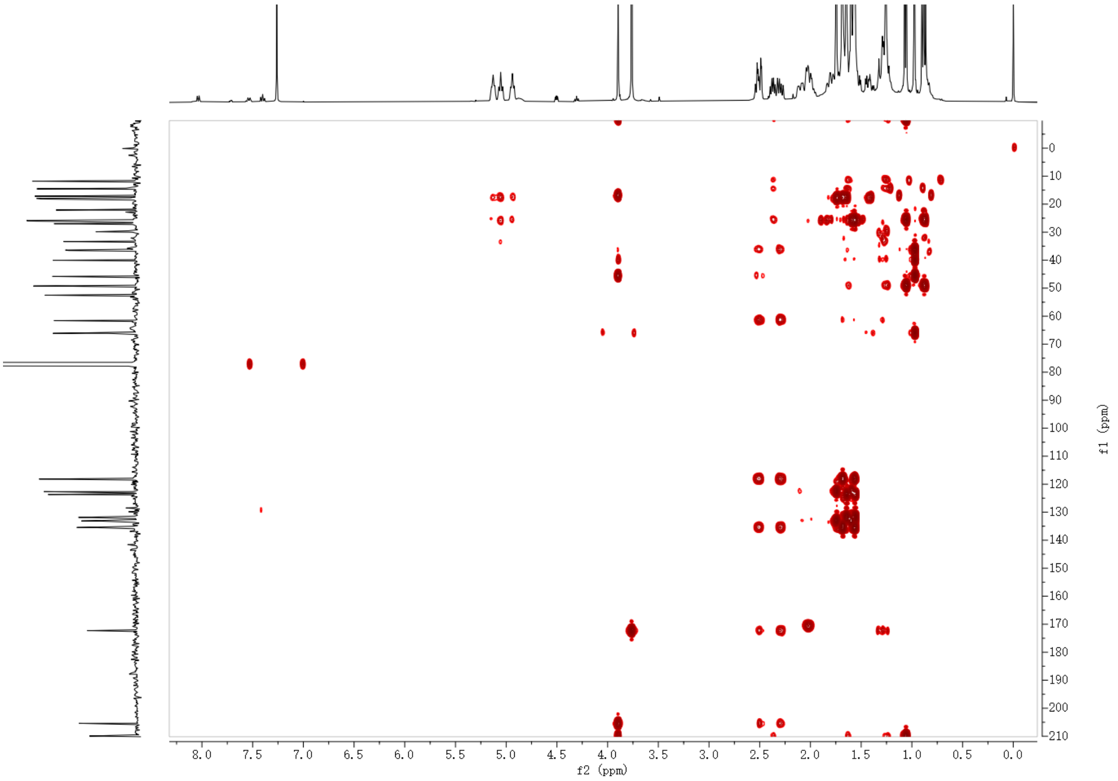

Figure S40. NOESY spectrum of **4** (400 MHz, $CDCl_3$)

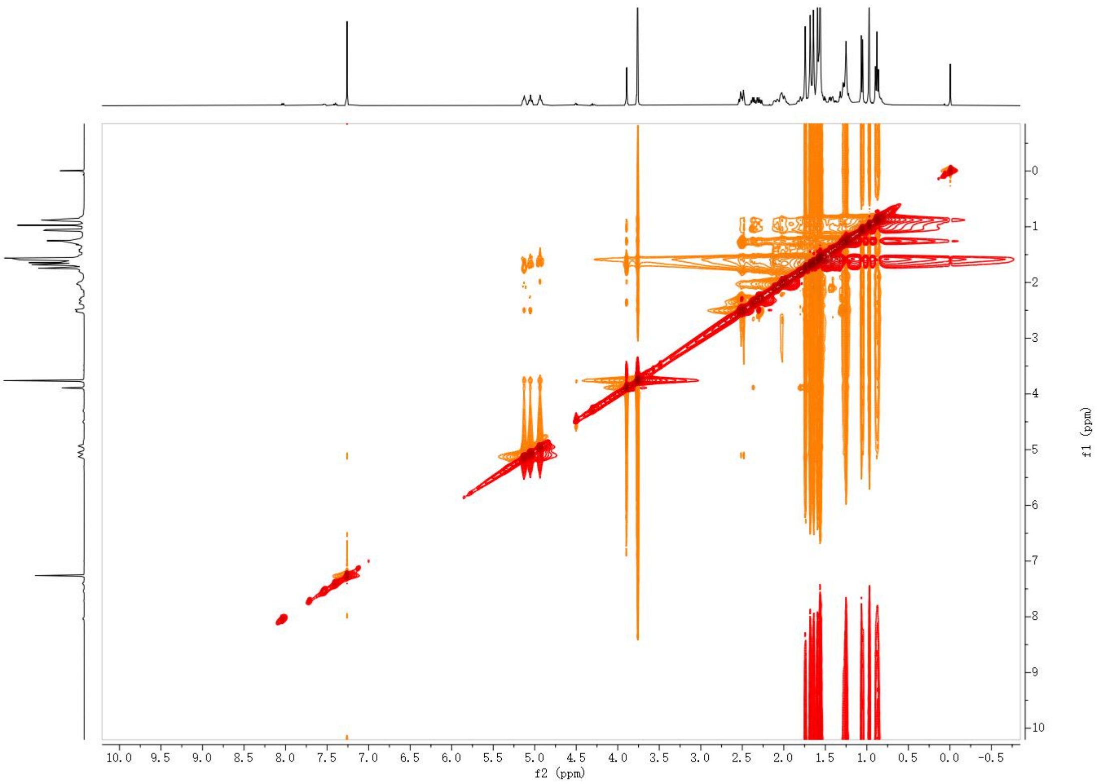

Figure S41. HR-ESI-MS spectrum of **4**

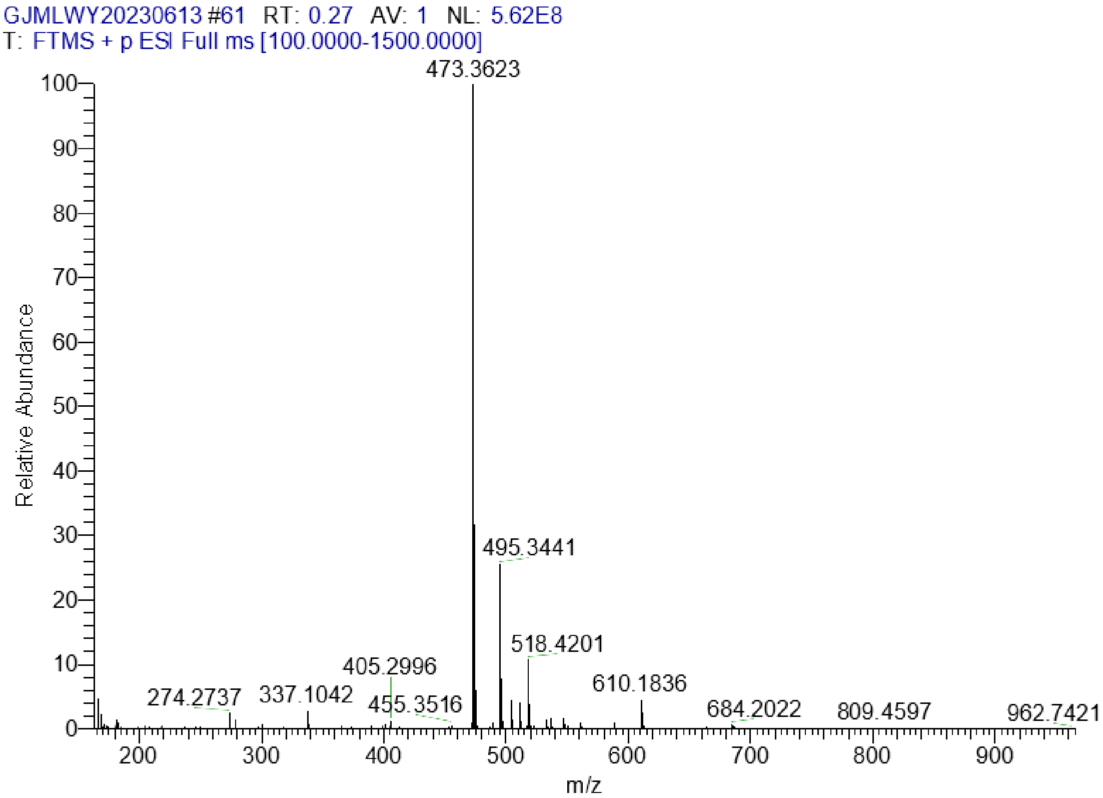

Figure S42. Experimental CD spectrum (in MeOH) of **4**

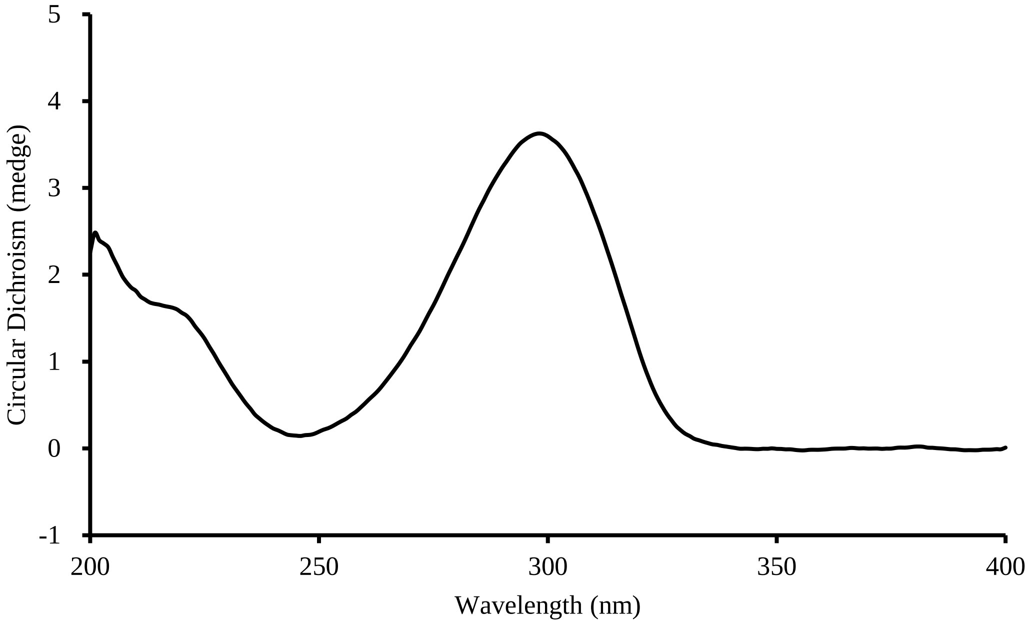

Figure S43. Experimental UV spectrum of **4**

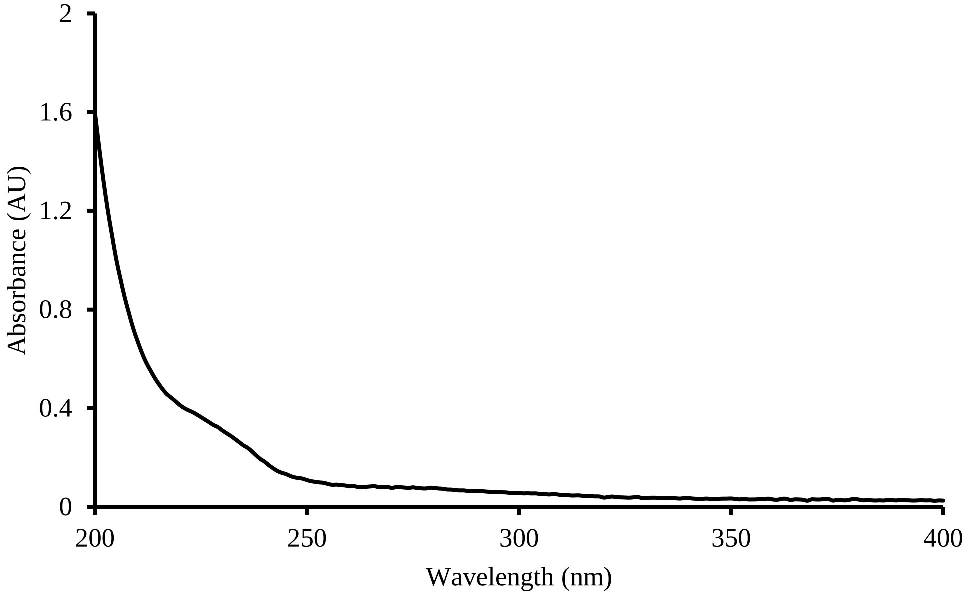

Figure S44. IR spectrum of **4**

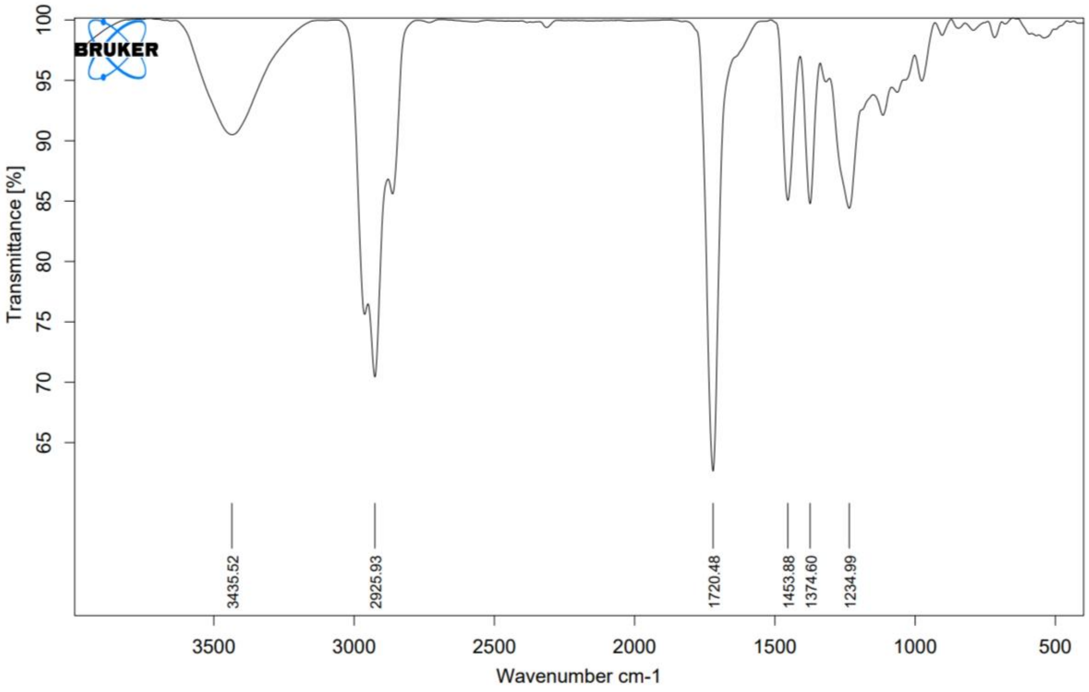

Spectra of physico-chemical properties of 5

Figure S45. ^{1}H NMR spectrum of **5** (400 MHz, $CDCl_3$)

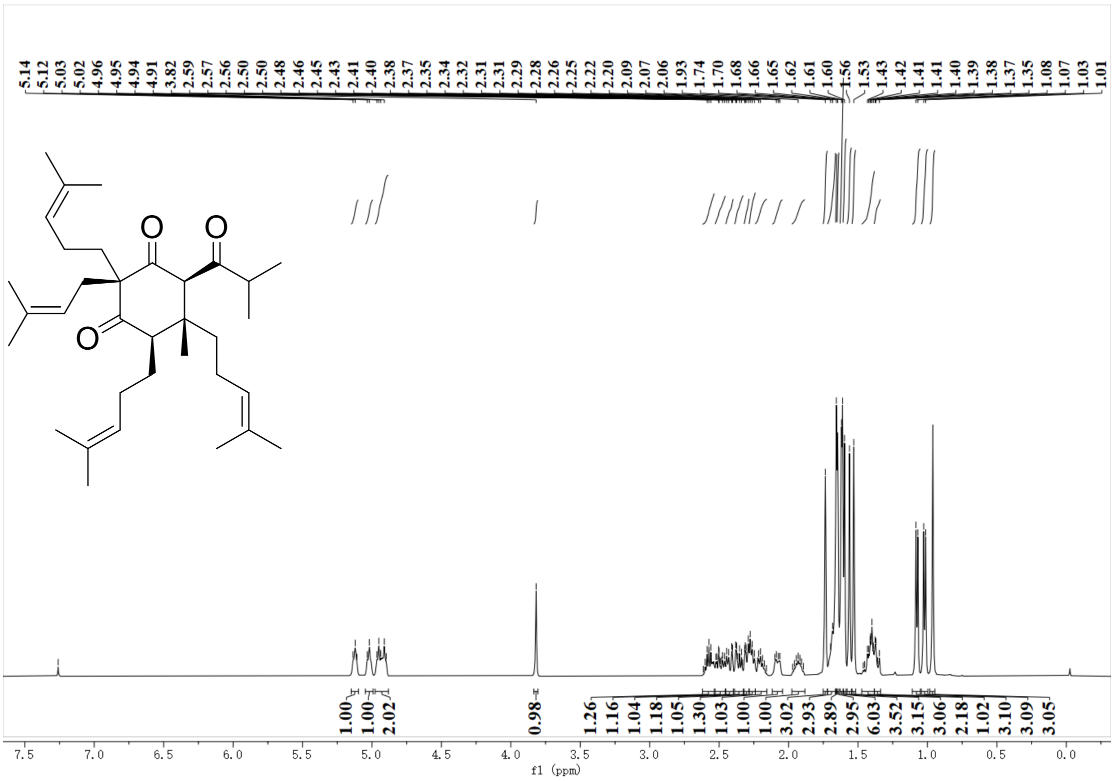

Figure S46. ^{13}C NMR spectrum of **5** (100 MHz, $CDCl_3$)

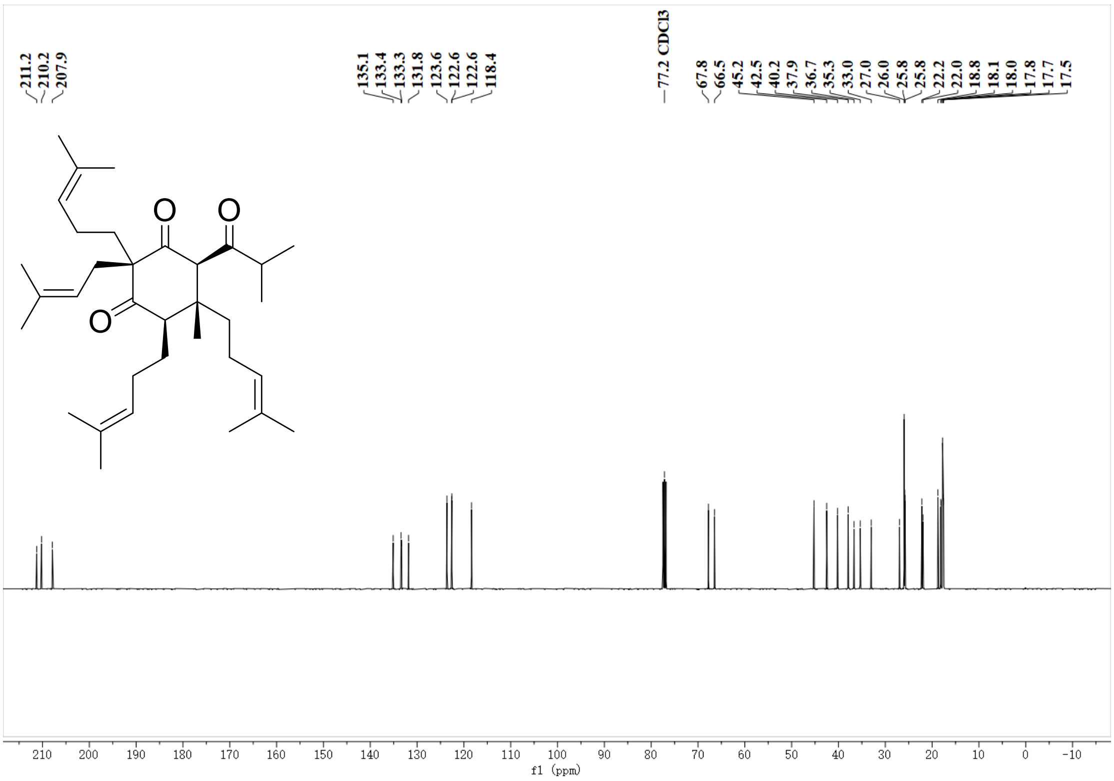

Figure S47. DEPT (θ = 135°) spectrum of **5** (100 MHz, $CDCl_3$)

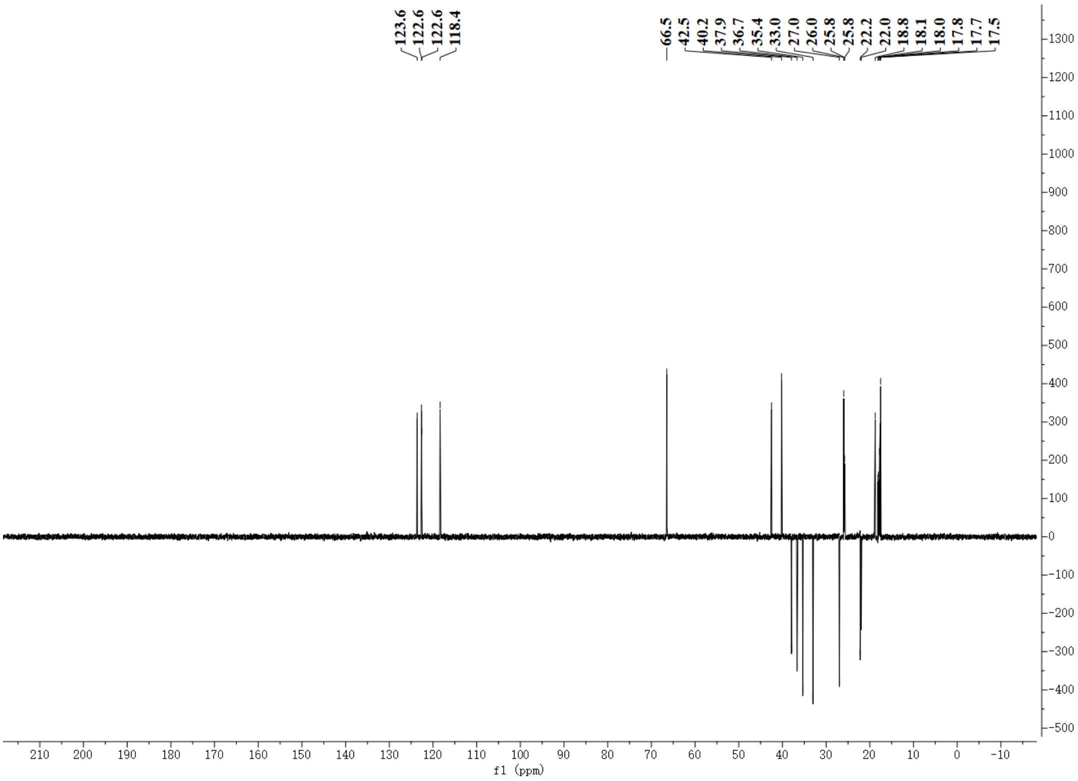

Figure S48. ^{1}H–^{1}H COSY spectrum of **5** (400 MHz, $CDCl_3$)

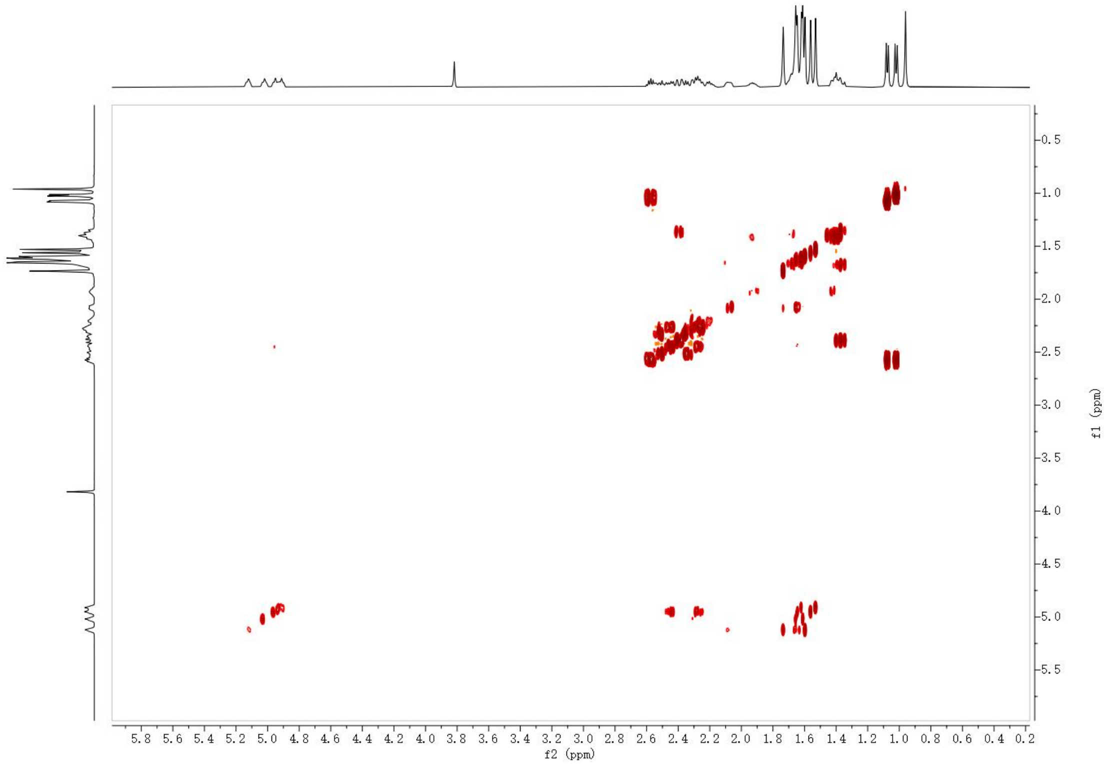

Figure S49. HSQC spectrum of **5** (400 MHz, $CDCl_3$)

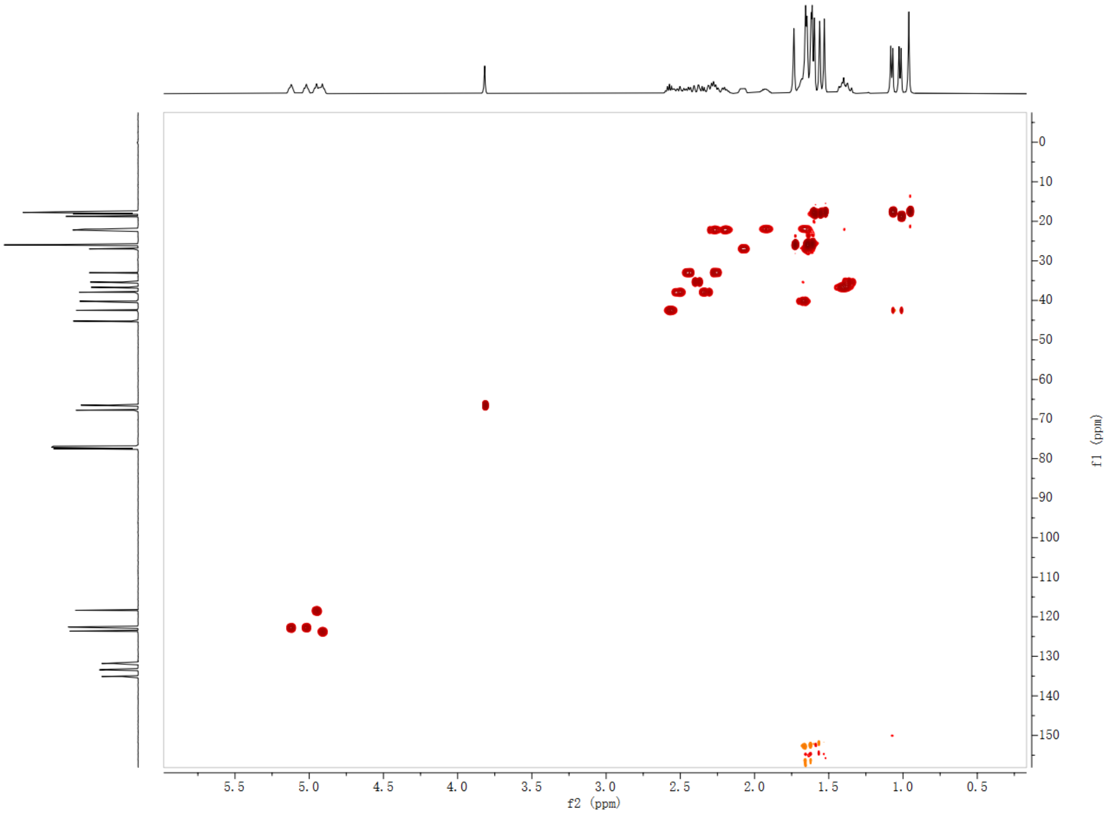

Figure S50. HMBC spectrum of **5** (400 MHz, $CDCl_3$)

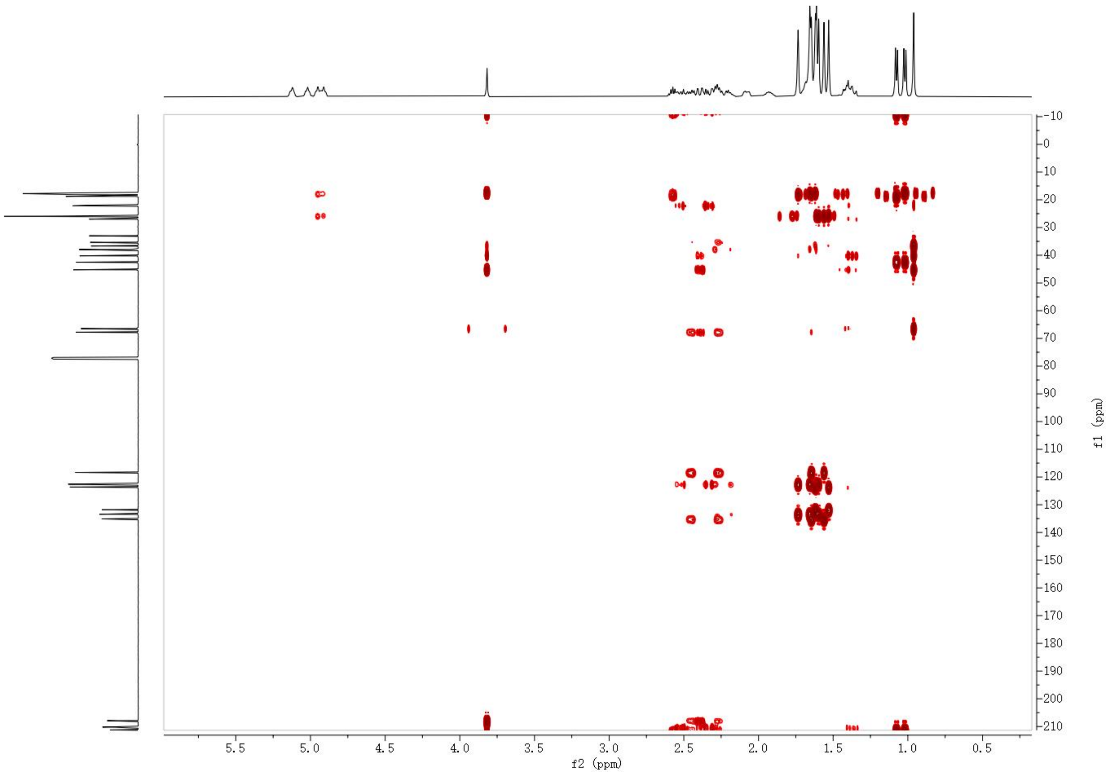

Figure S51. NOESY spectrum of **5** (400 MHz, $CDCl_3$)

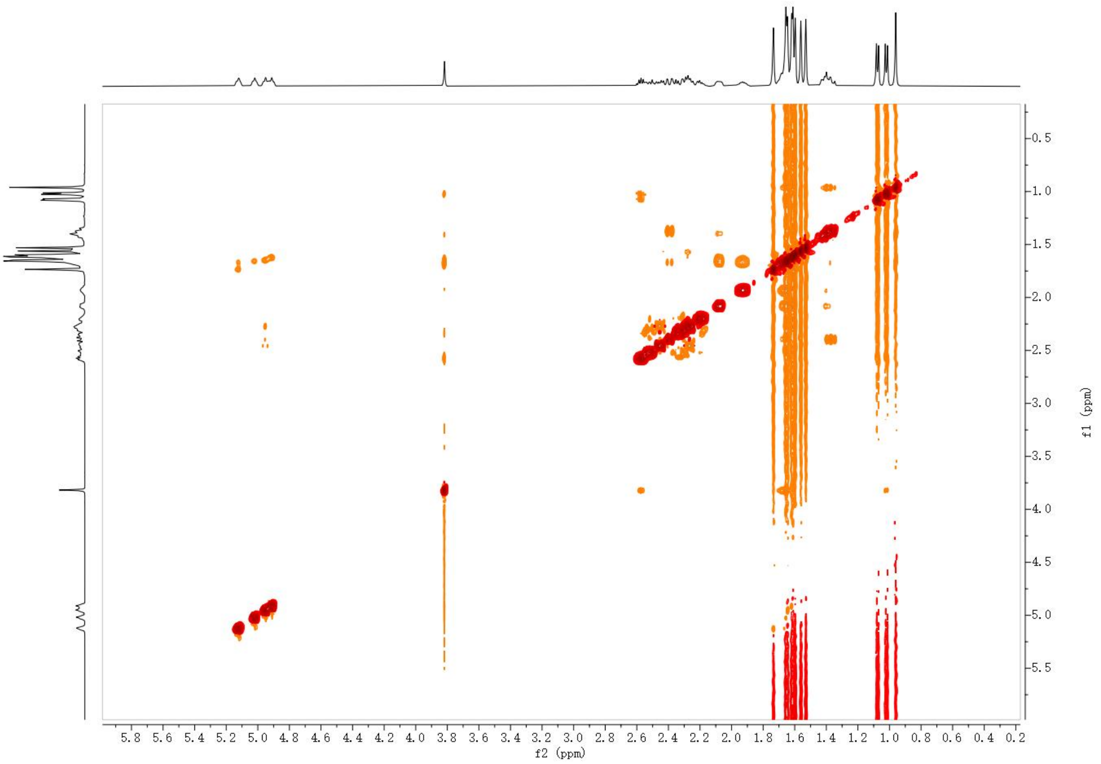

Figure S52. HR-ESI-MS spectrum of **5**

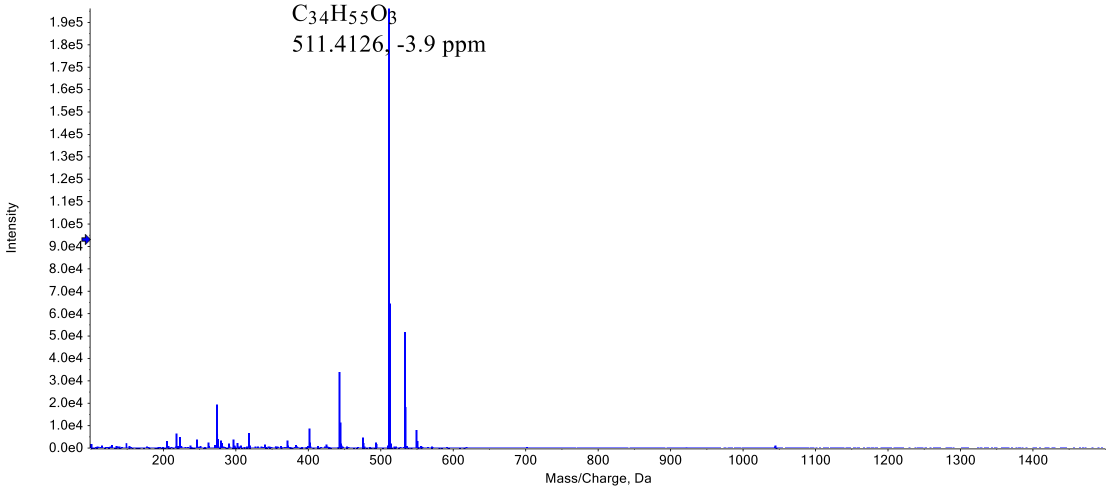

Figure S53. Experimental CD spectrum (in MeOH) of **5**

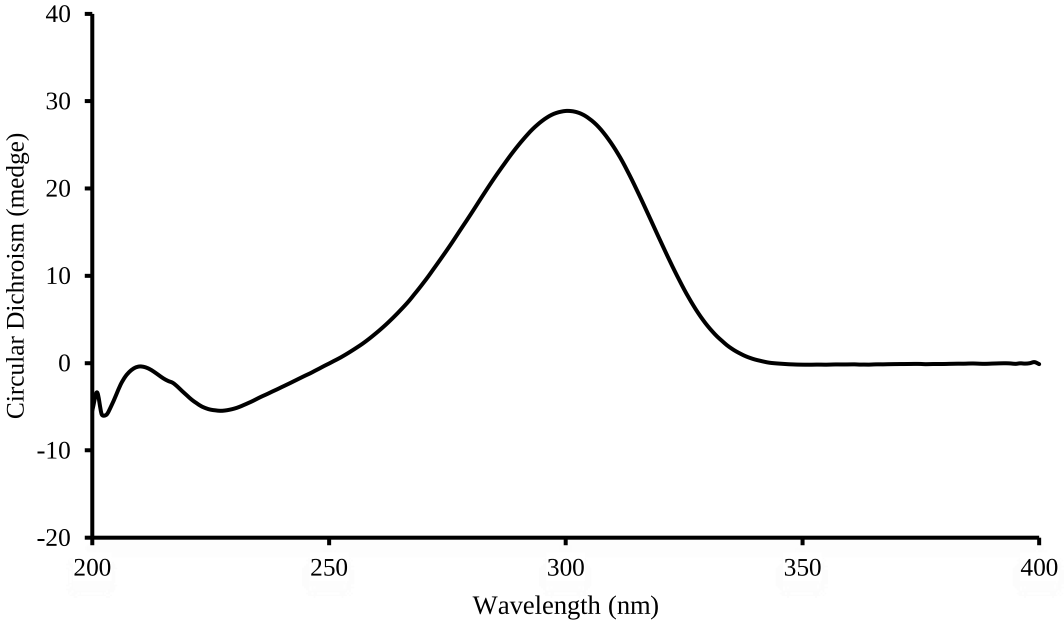

Figure S54. Experimental UV spectrum of **5**

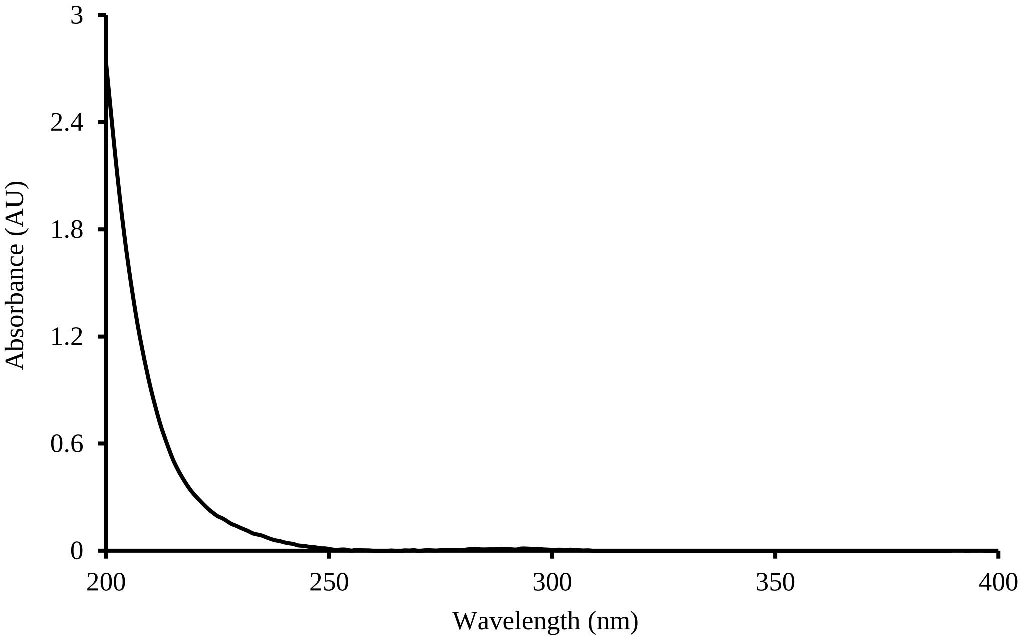

Figure S55. IR spectrum of **5**

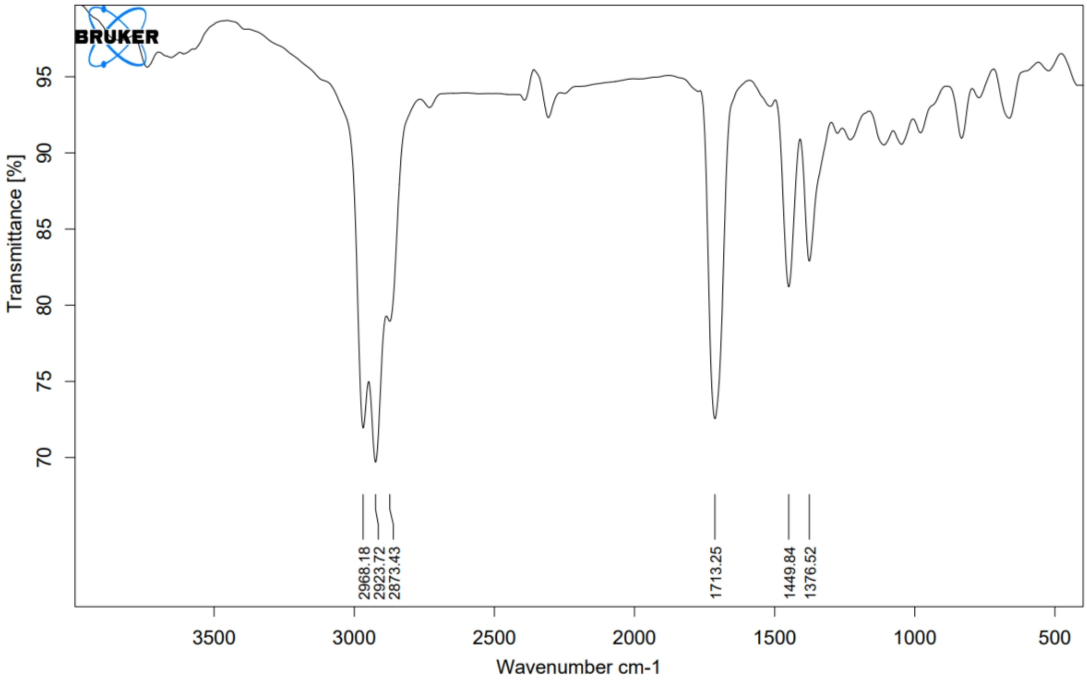

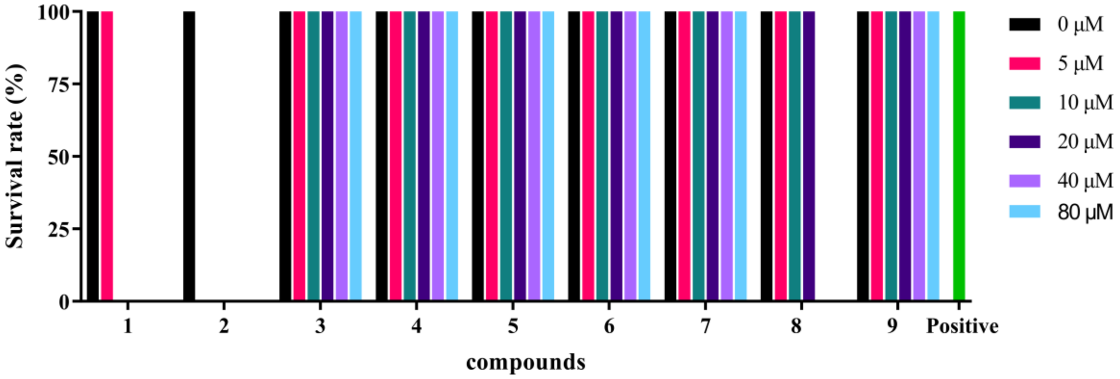

Figure S56. Results of toxicity tests of different compounds on zebrafish larvae (statistics at 7 dpf).

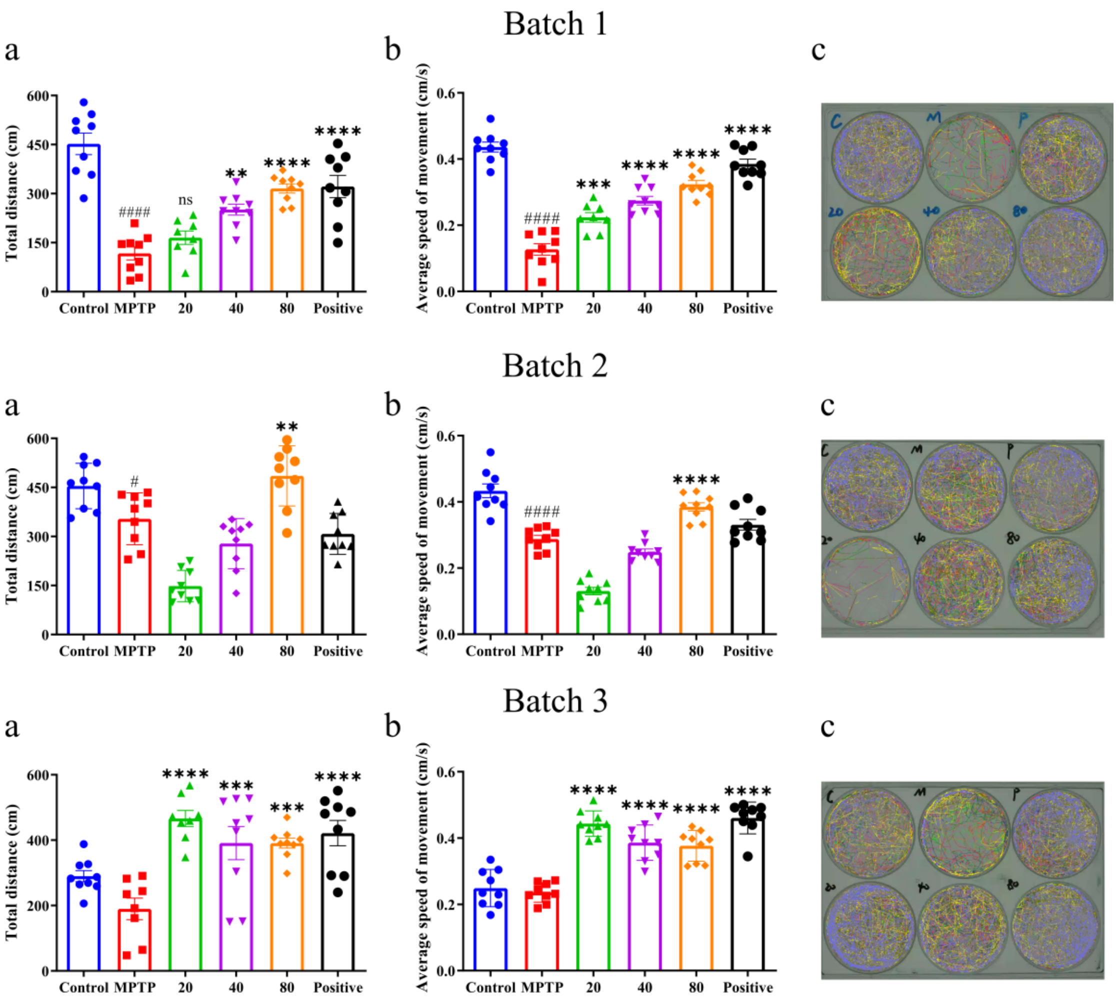

Figure S57. Effects of compound 9 on locomotor impairments induced by MPTP in three batches of zebrafish larvae. Total distance traveled (a), average speed (b), and track visualization image (c). The experimental groups included the control group, model group, treatment groups, and positive drug group (n = 8). #$p < 0.05$, ####$p < 0.0001$ vs. control (DMSO) group, **$p < 0.01$, ***$p < 0.001$, ****$p < 0.0001$ vs. MPTP group, and ns indicates no significance.
